# Metabolism of cancer cells commonly responds to irradiation by a transient early mitochondrial shutdown

**DOI:** 10.1016/j.isci.2021.103366

**Published:** 2021-10-28

**Authors:** Adam Krysztofiak, Klaudia Szymonowicz, Julian Hlouschek, Kexu Xiang, Christoph Waterkamp, Safa Larafa, Isabell Goetting, Silvia Vega-Rubin-de-Celis, Carsten Theiss, Veronika Matschke, Daniel Hoffmann, Verena Jendrossek, Johann Matschke

**Affiliations:** 1 Institute of Cell Biology (Cancer Research), University Hospital Essen, University of Duisburg-Essen, 45147 Essen, Germany; 2 Bioinformatics and Computational Biophysics, University of Duisburg-Essen, 45117 Essen, Germany; 3 Department of Cytology, Institute of Anatomy, Medical Faculty, Ruhr University Bochum, 44801 Bochum, Germany

**Keywords:** Mathematical biosciences, Cancer systems biology, Cancer

## Abstract

Cancer bioenergetics fuel processes necessary to maintain viability and growth under stress conditions. We hypothesized that cancer metabolism supports the repair of radiation-induced DNA double-stranded breaks (DSBs). We combined the systematic collection of metabolic and radiobiological data from a panel of irradiated cancer cell lines with mathematical modeling and identified a common metabolic response with impact on the DSB repair kinetics, including a mitochondrial shutdown followed by compensatory glycolysis and resumption of mitochondrial function. Combining ionizing radiation (IR) with inhibitors of the compensatory glycolysis or mitochondrial respiratory chain slowed mitochondrial recovery and DNA repair kinetics, offering an opportunity for therapeutic intervention. Mathematical modeling allowed us to generate new hypotheses on general and individual mechanisms of the radiation response with relevance to DNA repair and on metabolic vulnerabilities induced by cancer radiotherapy. These discoveries will guide future mechanistic studies for the discovery of metabolic targets for overcoming intrinsic or therapy-induced radioresistance.

## Highlights

• Mathematical modeling revealed common early mitochondrial shutdown upon irradiation
• Mitochondrial shutdown correlates to oxidizing effects of ionizing radiation
• Compensatory glycolysis fuels DNA repair during mitochondrial shutdown
• Oncogenic mutations impact mitochondrial recovery and DNA repair kinetics

## Graphical abstract

**Figure:**
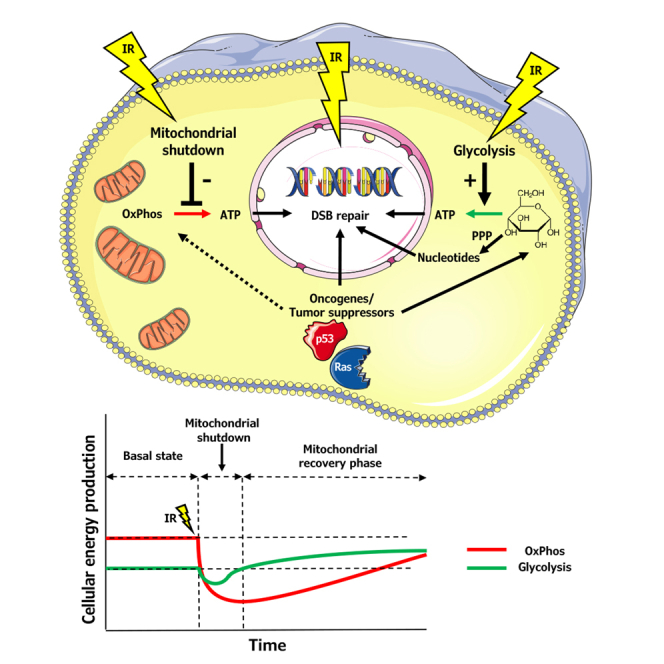

## Introduction

Altered cellular metabolism is an important feature of nearly all types of cancer and represents the key driving force for cancer growth and malignant progression (DeBerardinis and Chandel, 2016; Faubert et al., 2020; Hanahan and Weinberg, 2011). To date, several metabolic bottlenecks in a variety of tumors have been discovered. Glutamine and aspartate addiction are prominent examples of acquired tumor dependencies on metabolism for growth, metastasis, and therapy resistance (Alkan et al., 2018; Christen et al., 2016; Krall et al., 2016; Matschke et al., 2016; Son et al., 2013). Furthermore, mitochondrial oxidative phosphorylation (mitochondrial respiration) and its extended function toward production of non-essential amino acids, like aspartate; nucleotide synthesis; and balancing the intracellular reduction equivalents/redox homeostasis are considered to be of major importance for cancer cell survival, invasion, and metastasis (Chandel, 2015; Elia et al., 2018; Faubert et al., 2020; Lozoya et al., 2018). The ability of cancer cells to adapt their metabolism to cope with stress conditions in the surrounding environment, the extent of their flexibility to use different fuels, as well as the metabolic heterogeneity of cells within a tumor are considered to be major determinants of resistance to anticancer treatment (Morandi and Indraccolo, 2017). Consequently, targeting these determinants is a promising approach to hit such tumor addiction nodes. Major challenges of using metabolic inhibitors in the field of cancer therapy remain the pronounced metabolic plasticity of cancer cells and the large molecular heterogeneity within and between different tumors, highlighting the urgent need for developing reliable biomarkers for precise patient stratification (Boudreau et al., 2016; Shackelford et al., 2013; Yuneva et al., 2012). In the field of precision cancer medicine, tumor metabolism offers a wide spectrum of modern omics methods—metabolomics, transcriptomics, and other –omics—as well as bioinformatic tools for the discovery of biomarkers. So far, the focus was on identifying and targeting metabolic dependency mechanisms and genetic constellations fueling the growth of specific types of cancer (Bellier et al., 2020; Shackelford et al., 2013). Besides these approaches, our group and others explored metabolic dependencies induced by adverse conditions in the tumor microenvironment (Matschke et al., 2016; Sonveaux et al., 2008). For example, metabolic reprogramming in response to cycling severe hypoxia-reoxygenation stress rendered hypoxia-reoxygenation-tolerant cancer cells more resistant to the cytotoxicity of ionizing radiation (IR) and also created metabolic vulnerabilities for a targeted radiosensitization and for overcoming acquired radioresistance (Hlouschek et al., 2018a, 2018b; Matschke et al., 2016; Rouschop et al., 2013). We therefore hypothesized that unraveling adaptive metabolic changes of cancer cells exposed to chemotherapy and/or radiotherapy will help to identify further novel and specific targets for combination therapy and to overcome therapy resistance caused by metabolic adaptation.

DNA repair demands high energy inputs, which potentially links cancer cell metabolism and resistance to irradiation (Götting et al., 2020; Rashmi et al., 2018; Tang et al., 2018; Turgeon et al., 2018). The genetic and metabolic capacity of cells to repair radiation-induced lethal DNA lesions with high efficiency is considered as one critical determinant of radiation resistance (Mladenov et al., 2013; Roos et al., 2015). However, little is known about differences in the response of cancer cells with distinct genetic backgrounds and specific metabolic phenotypes on the early cellular response to IR. This prompted us, first, to use extracellular flux analyses to systematically screen a panel of nine cancer cell lines of different origins, distinct genetic backgrounds, and mutational loads for their acute metabolic response to IR, and second, to correlate their ability to recover from IR-induced cell stress to the kinetics of DNA repair (Pouliliou and Koukourakis, 2014). Despite significant dissimilarities of the screened cancer cell lines in genetic background and basal metabolic state, we observed a common early, transient downregulation of glycolysis and a surprising mitochondrial inactivation shortly after exposure to IR, which we termed a “mitochondrial shutdown.” This shutdown was followed by a fast recovery of glycolytic capacity and by a slow, uniform, and gradual reconstitution of mitochondrial respiration, irrespective of genetic background and mutational load. However, the genetic background appeared to be an important factor for the kinetics and efficiency in the repair of IR-induced DNA damage, for the use of glycolytic pathways to maintain metabolic homeostasis, and thus potentially for the metabolic abilities of the cells to fuel DNA repair. We conclude that IR by its general physical oxidizing effects blocks mitochondrial respiration independent of genetic background, thereby offering a window for therapeutic intervention. Normal cells also underwent mitochondrial shutdown in response to IR but recovered faster from initial functional inactivation, highlighting critical differences between physiological and altered cancer metabolism.

Thus, combining IR with inhibitors of mitochondrial respiration or compensatory glycolysis may be well suited to specifically increase cytotoxic effects of IR in cancer cells and thereby potentially enhance the efficacy of cancer radiotherapy.

## Results

### Cancer cell lines are characterized by high heterogeneity in metabolic activity and phenotype

**Figure 1:**
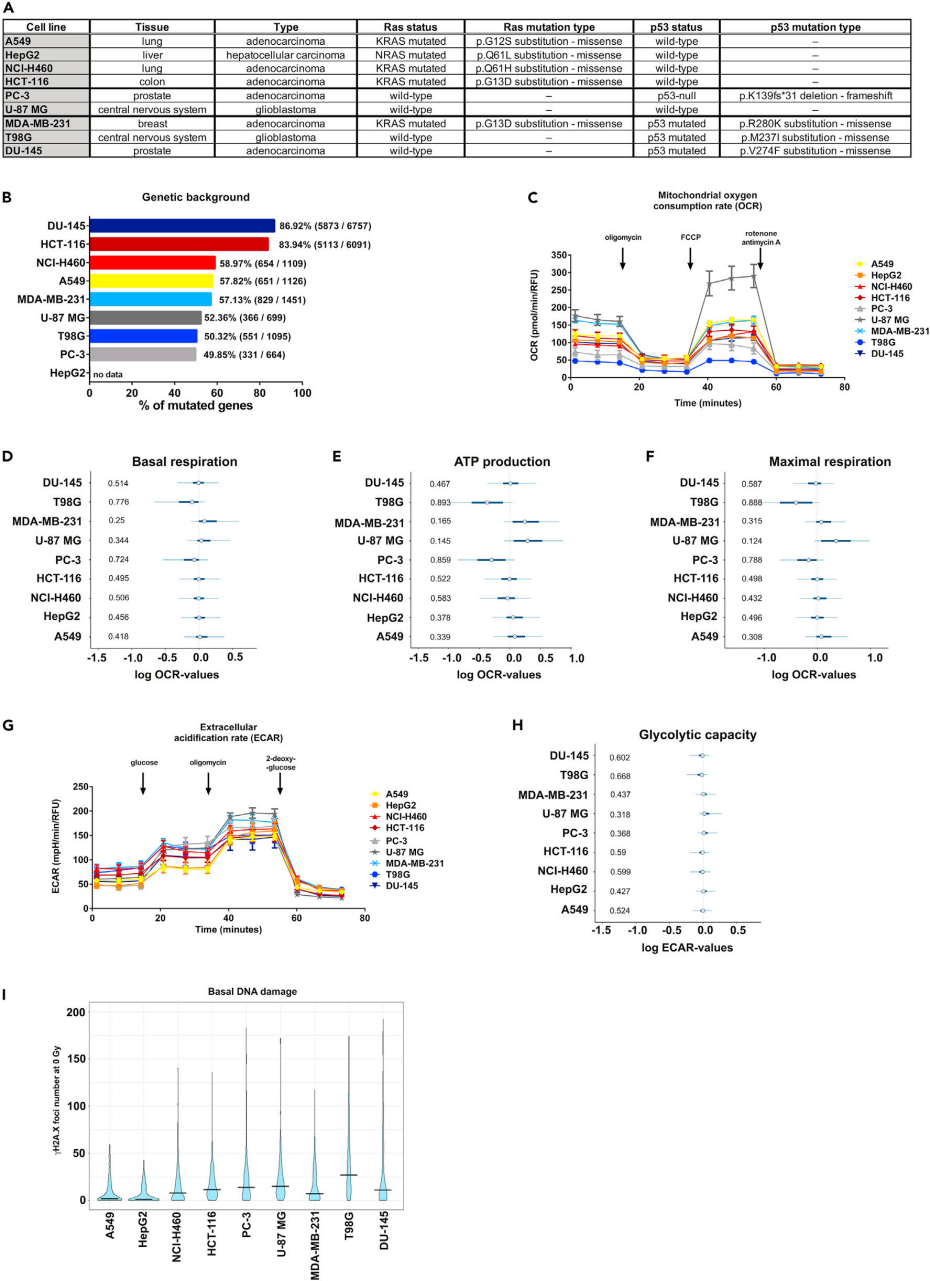
Diverse cancer cell lines differ in basal metabolic activity, mutational burden, and endogenous level of DNA double-stranded breaks (A) Table summarizing origin, type, and mutational status of Ras and p53 of the screened cancer cell lines. (B) Mutational burden of screened cancer cell lines depicted as a percentage of mutated genes of total number of genes analyzed. (C) Metabolic activity in a panel of nine different cancer cell lines expressed as mitochondrial oxygen consumption rate related to oxidative phosphorylation. Mean values ± SEM are plotted. (D–F) Effect of the respective cancer cell line on logarithmic basal respiration (D), mitochondrial ATP production (E), and maximal respiration (F) relative to corresponding average over all tested cell lines. Blue circles are median effects; bold and thin lines mark 50% and 95% of highest probability density (“credible intervals”), respectively. Numbers on the left are probabilities P₋ of negative effects, i.e., probabilities of log values being lower than the average of all cell lines (average marked by thin vertical lines). Each value characterizes oxygen consumption rate (OCR) obtained from n = 8–16 wells for each cell line, combined from N = 2 independent experiments and was normalized to cell number at the end of the assay (pmol/min/RFU). (G) Glycolysis stress test performed to determine parameters of glycolysis (Figure S1F), glycolytic capacity (H), and non-glycolytic acidification (Figure S1H) in a panel of nine different cancer cell lines. Mean values ± SEM are plotted. (H) Effect of cell line on logarithmic glycolytic capacity with respect to average glycolytic capacity of all tested cell lines. (I) Basal DNA damage levels indicated as the number of all measured γH2A.X foci in non-irradiated cells without excluding damaged or stressed cell nuclei. Violins show distributions of all measured foci numbers per cell without excluding damaged or stressed cell nuclei from at least 50 nuclei per experiment, of N = 3 independent biological replicates. Horizontal bars mark medians. See also Figure S1.

First, we analyzed metabolic parameters and energy expenditures of nine well-characterized cancer cell lines with diverse origins and with different genetic backgrounds (A549, HepG2, NCI-H460, HCT-116, PC-3, U-87 MG, MDA-MB-231, T98G, DU-145; see Figures 1A and 1B) under standard culture conditions, without IR treatment. For this purpose, two parameters of cellular energetics were measured in real time: oxygen consumption rate (OCR, associated with mitochondrial respiration and oxidative phosphorylation) and extracellular acidification rate (ECAR, related to glycolytic activity of cells). Further specific metabolic parameters, such as mitochondrial ATP production, were also assessed with inhibitor-induced changes. As expected, cancer cell lines displayed a range of mitochondrial functionality (Figure 1C). Remarkably, the credible intervals for basal mitochondrial respiration (Figures 1D and S1) and ATP production (Figures 1E and S1) of breast cancer MDA-MB-231 and glioblastoma U-87 MG cell lines had tendencies toward higher values, consistent with their elevated basal mitochondrial respiration (Figure 1C). Mitochondrial uncoupling by carbonyl cyanide-4-(trifluoromethoxy) phenylhydrazone (FCCP) forced the respiratory chain to operate at its maximal rate, i.e., at maximal oxygen consumption. Consequently, the credible intervals for maximal respiration rates of the most metabolically active cell lines, MDA-MB-231 and U-87 MG, again had tendencies toward higher values (Figures 1F and S1). ECAR values, representing the use of the glycolytic pathway, also varied between cell lines, although to a lesser extent than the mitochondrial metabolism (Figures 1G, 1H, and S1). To further elucidate glucose metabolism, we performed a glycolysis stress test and thereby revealed distinct capabilities of cells to acutely use glucose when mitochondrial respiration is blocked (Figures 1H, S1F, and S1H). Finally, analysis of basal DNA damage demonstrated by γH2A.X foci largely overlapped across tested cell lines (Figure 1I). However, higher metabolic activity was not directly associated with the level of mutational burden of the screened cells obtained from the COSMIC Cell Line Project (cancer.sanger.ac.uk) (Figures 1A and 1B) (Sondka et al., 2018).

### Ionizing radiation acutely lowers mitochondrial metabolic activity of cancer cells

**Figure 2:**
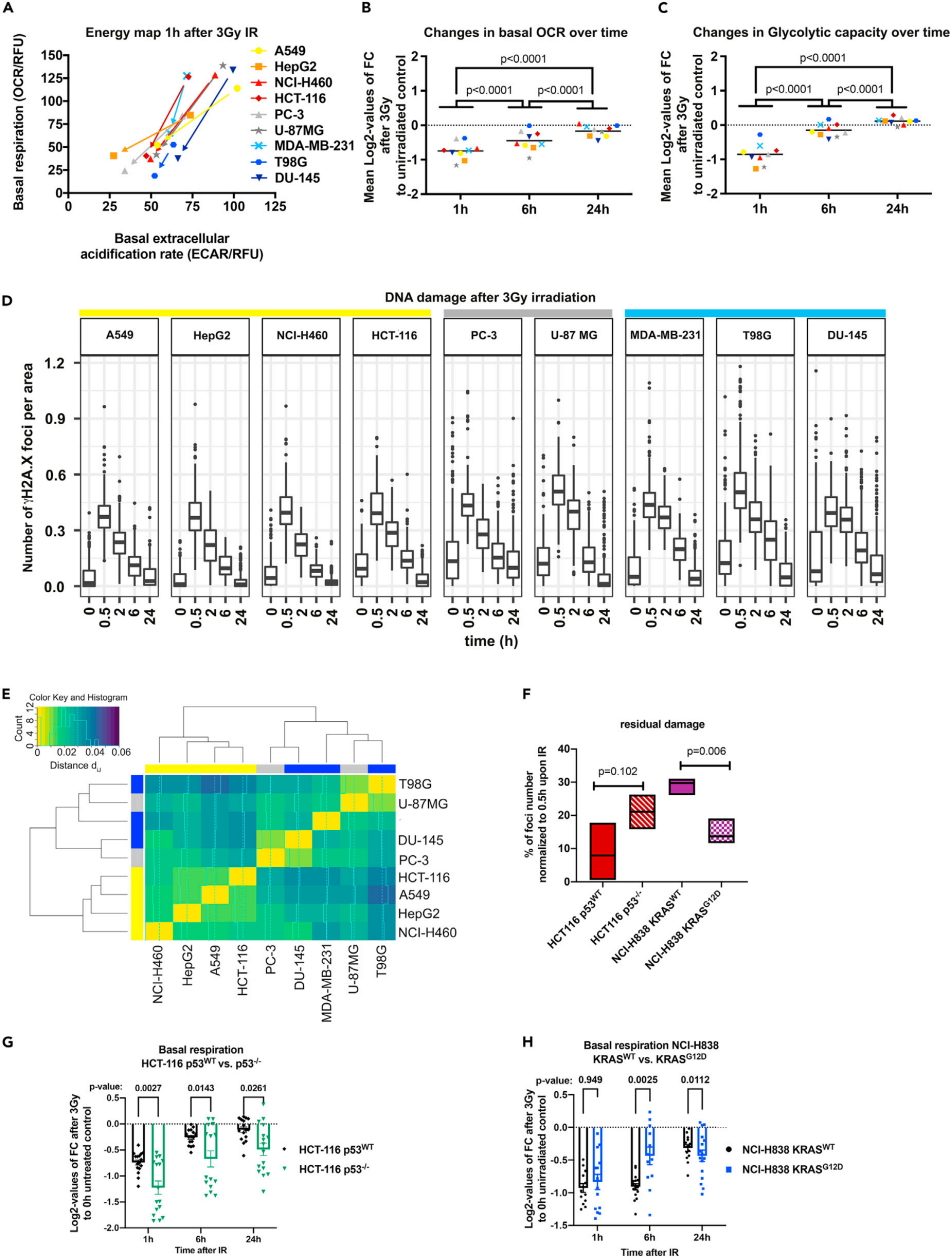
Exposure to ionizing radiation (IR) acutely lowers metabolic activity of cancer cells (A) Energy map of the cancer cell line panel indicating levels of basal mitochondrial respiration (OCR, vertical axis) and extracellular acidification rate (ECAR), associated with glycolytic activity (horizontal axis) before and 1 h after exposure to IR with a dose of 3 Gy. Arrows indicate the change in measured OCR and ECAR parameters at 1 h after IR compared to non-irradiated control for each cancer cell line. (B and C) Log2-values of changes in basal respiration (B) and glycolytic capacity (C) induced by IR with a dose of 3 Gy or basal respiration compared with non-irradiated controls at 1, 6, and 24 h after IR. Mean values of n = 12–16 wells per cell line from N = 2 independent experiments are indicated. All metabolic parameters were normalized to Hoechst intensity (relative fluorescence units, RFU) in each well. OCR, oxygen consumption rate; ECAR, extracellular acidification rate; FC, fold change; p values calculated using two-way ANOVA with Tukey's multiple comparisons post hoc test. (D) Foci number per area upon 3 Gy of all cell lines between 0 and 24 h divided in Ras- or p53-mutated. Data represent the counted raw values used for normalization in Figures S3D and S3E. Yellow, cell lines with Ras mutation; gray, intermixed mutation; and blue, p53 mutations. (E) Heatmap of hierarchically clustered distances (d_ij_) of γH2A.X foci removal over all time points showing distance d_ij_ of DSB time course vectors of cell lines i and j. Groups are marked as follows: yellow, Ras mutation, p53^WT^; gray, intermixed mutation; blue, p53 mutation, Ras^WT^ (see Figure S4 for posterior predictive check demonstrating the validity of the statistical model used in (E) and for expected number of γH2A.X foci at indicated time points in all cell lines). (F) Residual DNA damage represented as % of γH2A.X foci number at the 24 h time point normalized to 30 min time point. Mean values ± SEM (N = 3) are plotted. p values calculated using two-way ANOVA with Tukey’s multiple comparisons post hoc test. (G and H) Log2-values of changes to measured basal respiration induced by IR with a dose of 3 Gy compared with non-irradiated controls 1, 6, and 24 h after treatment indicated ((G) HCT-116 p53^WT^ versus HCT-116 p53^−/−^; (H) NCI-H838 KRAS^WT^ versus NCI-H838 KRAS^G12D^); mean values of n = 12–16 wells per cell line from N = 2 independent experiments are indicated ± SEM (p values calculated using two-way ANOVA with Šídák's multiple comparisons post hoc test). See also Figures S2–S5 and S8.

We next examined the acute effects of IR on cell metabolism at a single dose of 3 Gy. Remarkably, we observed a general reduction of mitochondrial respiration and ECAR in all cell lines. However, the most pronounced effect was noticed 1 h after exposure to IR (Figure 2A). In addition, mitochondrial respiration, ATP production, and forced maximal respiration after mitochondrial uncoupling were strongly decreased 1 h after IR, resulting in a mitochondrial inactivation, a phenomenon that we termed a mitochondrial shutdown (Figures 2B, S2A, S3A, and S3B). Mitochondrial respiration did not bounce completely back over 24 h from its lowest point after 1 h toward levels of non-irradiated controls (Figures 2B, S2A, S3A, and S3B). Of interest, we observed a low initial glycolytic activity (ECAR, Figures 2A and S2) and glycolytic capacity 1 h after IR and almost complete recovery of glycolytic activity 6 h after IR in all tested cell lines (Figures 2C, S2A, and S3C). The capability to recover mitochondrial respiration toward the levels of non-irradiated controls within 24 h after 3 Gy of IR has been observed across all cell lines (Figures 2B, S3A, and S3B). It is noteworthy that the recovery of mitochondrial activity was not complete in terms of mitochondrial metabolic parameters in any of the cell lines (Figures 2B, S2A, S3A, and S3B). In contrast, the glycolytic capacity had been recovered to the basal levels 6 h post IR, and for some cell lines it even reached higher levels than the non-irradiated controls after 24 h (Figures 2C, S2A, and S3C). As a control we also examined the effects of radiation on mitochondrial metabolism in normal lung epithelial cells (HSAEC1-KT) and observed an early and transient IR-induced mitochondrial shutdown in these cells (Figure S5E). However, in contrast to the cancer cells the recovery of basal mitochondrial respiration from initial IR-induced mitochondrial shutdown in normal epithelial cells reached levels of non-irradiated controls already after 6 h (Figure S5E). These results hint at significant differences in the ability between tumor and normal tissue cells to cope with therapy-induced metabolic stress, particularly to recover from IR-induced mitochondrial shutdown. In summary, we observed a common early and transient decrease in mitochondrial function, which we termed mitochondrial shutdown. However, the recovery from IR-induced mitochondrial shutdown differed between tumor and normal cell lines and depended on the genetic background (Figures 2G, 2H, and S5C–S5E). Yet, further investigations are needed to reveal the potential impact of early mitochondrial shutdown on the survival of irradiated cancer cells (Figures S3H and S3I).

### The genetic background impacts DNA repair kinetics in irradiated cancer cells

To assess a possible association between the cell metabolism and the efficiency in the repair of IR-induced DNA double-stranded breaks (DSBs), we monitored the kinetics of removal of γH2A.X foci at 30 min, 2 h, 6 h, and 24 h after IR (Pouliliou and Koukourakis, 2014). Based on the rate of γH2A.X foci removal (Figures 2D, S3D, and S3E), we subdivided the cell lines into a fast (A549, NCI-H460, HepG2, and HCT-116; yellow, Figure S3D), intermediate (PC3, U87-MG; gray, Figure S3D), and a slow (T98G, DU-145, and MDA-MB-231; blue, Figure S3D; Figure S3E) repairing group. Between these groups, we observed the most pronounced differences in DNA repair kinetics when comparing foci numbers at 0.5–2 h for A549, HepG2, NCI-H460, and T98G and at 2 and 6 h for the other cell lines after IR (Figure S3G). Here, U87-MG displayed the second highest level of γH2A.X, which correlated with an insufficient G2 arrest in the response to IR (Figure S8I).

Statistical analysis of γH2A.X foci removal over time revealed a correlation between genetic background and DNA repair kinetics (Figures S3D and S4). These results implicate that the speed of γH2A.X foci removal depends on the genetic background, particularly on the oncogenic regulators Ras and p53. Here, rapid removal of IR-induced γH2A.X foci correlated to mutated Ras, whereas Ras^WT^ cell lines were characterized by slower DNA repair kinetics (Figures 2E and S4). In detail, the MDA-MB-231 breast cancer cell line harboring mutations in both KRAS and p53 had delayed DNA repair kinetics. However, delayed DNA repair kinetics were also noticed in prostate PC-3 cells with p53^−/−^ and KRAS^WT^ and in prostate DU-145 with p53^V274F^. These findings confirm a strong heterogeneity and multifactorial genetic control of DSB repair kinetics across cancer cells corroborating those genetic alterations beyond oncogenic drivers will influence the observed responses (Figures 2D and S4).

To decipher the direct genetic influence of mutations in Ras or p53 on the observed phenotypes of DSB repair kinetics upon IR we used isogenic cell lines harboring specific mutations in Ras (NCI-H838 KRAS^WT^ and NCI-H838 KRAS^G12D^) or p53 (HCT-116 p53^WT^ and HCT-116 p53^−/−^). Here, the occurrence of a KRAS mutation correlated with both an increased removal of IR-induced γH2A.X foci (Figures 2F and S5B) and accelerated recovery from IR-induced mitochondrial shutdown (Figures 2H and S5D). By contrast, mutations in p53 potently slowed DNA repair kinetics (Figures 2F and S5A) and attenuated the recovery of mitochondrial function from IR-induced mitochondrial shutdown (Figures 2G and S5C). Consequently, our data suggest an opposite influence of mutant KRAS or p53 defects on DNA repair kinetics and recovery from IR-induced mitochondrial function.

### IR-induced metabolic activity and DNA DSB repair correlate in a time-dependent manner

**Figure 3:**
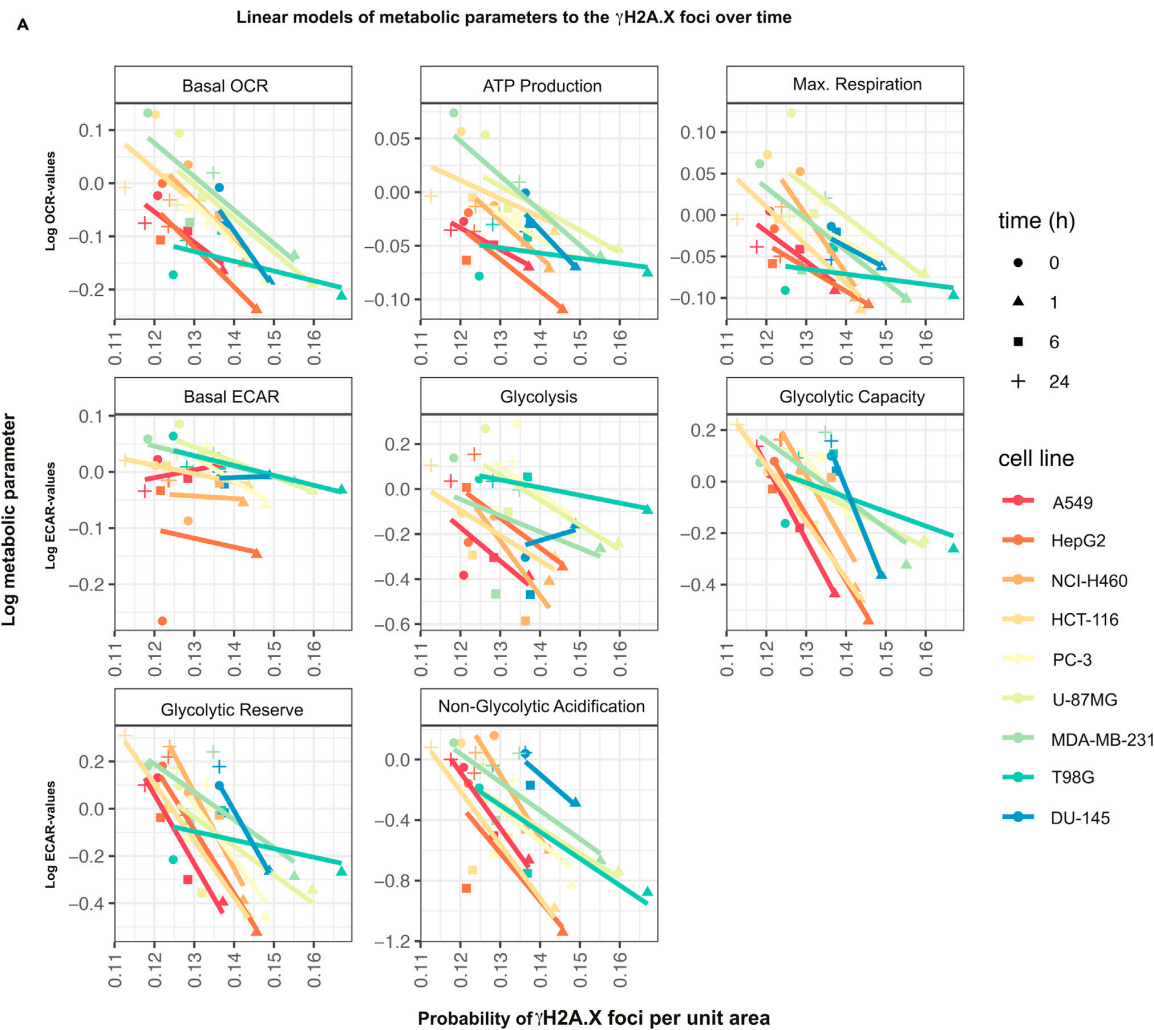
Linear models of time courses of metabolic parameters and DNA damage response Linear models of probabilities of γH2A.X foci per unit area (0.1∗Pixel^2^) and log-metabolic parameter at four time points for different tumor cell lines. Linear models revealed a strong negative correlation (except for the basal ECAR) of IR-induced time-dependent alterations in metabolic parameters with the resolution of γH2A.X foci with a similar pattern (for slopes of lines see Figure S7A). See also Figures S6 and S7.

To gain insight into a potential association between DSB repair kinetics and metabolic parameters, we correlated the recovery of the mitochondrial respiration and glycolysis parameters within the 24 h after IR (3 Gy) to the progression of γH2A.X foci removal over time. To this end, we computed linear models relating metabolic parameters and probabilities of γH2A.X foci per unit area at four different time points (Figures 3 and S6A). This analysis revealed that overall IR-induced alterations in metabolic parameters negatively correlated with the removal of γH2A.X foci for all tested cell lines, irrespective of the underlying Ras or p53 mutations (Figures 3 and S7A). A notable exception of this negative correlation was basal ECAR, where slopes varied around zero. Taken together, our results indicate that, across different cancer cell lines, exposure to IR induces a severe metabolic stress that decreases as γH2A.X foci are removed (Figures S7A and S7B). Our results reveal a two-way interconnection of cellular metabolism and DNA repair pathways, offering a starting point for further mechanistic investigations.

### Mitochondrial shutdown correlates with IR-induced physical oxidizing effects

**Figure 4:**
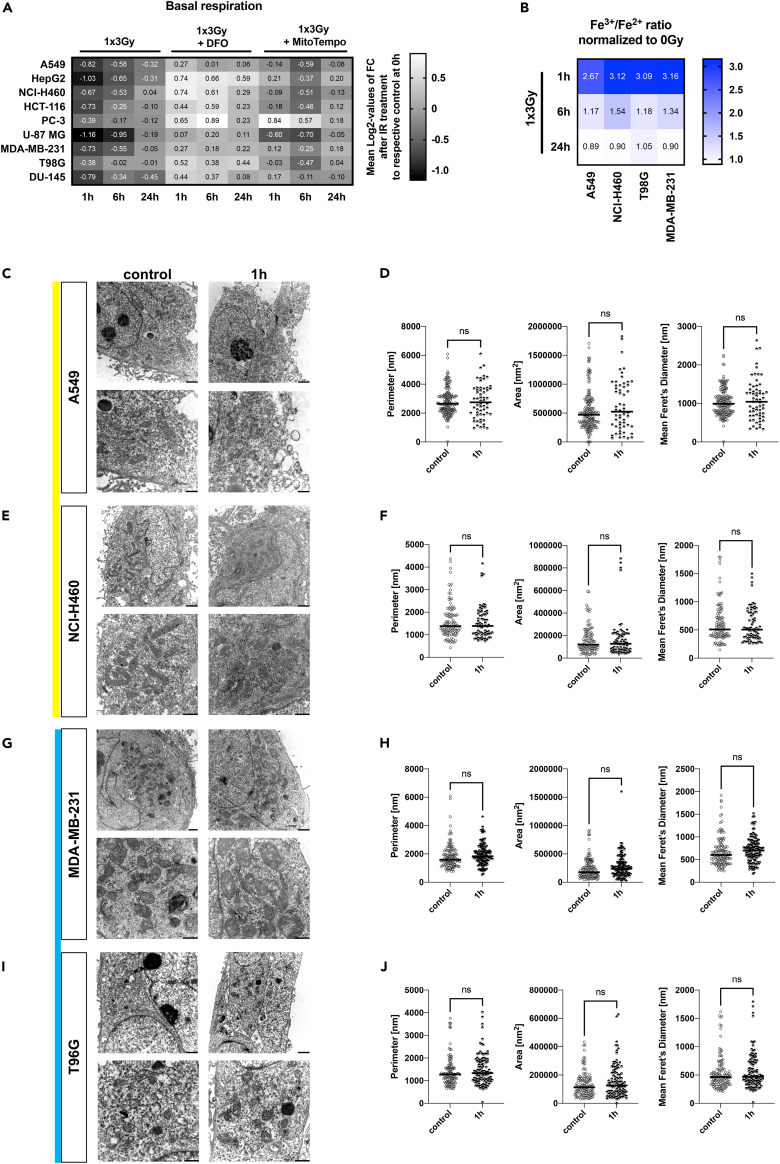
Mitochondrial shutdown correlates with IR-induced oxidizing effects (A) Mean Log2-values of changes to measured basal respiration induced by IR alone or in combination treatment with DFO (50 μM) or MitoTempo (1 nM) with a dose of 3 Gy compared with non-irradiated controls 1, 6, and 24 h after treatment. (B) Mean Fe^3+^/Fe^2+^ ratios induced by IR with a dose of 3 Gy 1, 6, and 24 h after IR normalized to non-irradiated controls. For calculated Fe^3+^/Fe^2+^ ratios ±SD induced by IR with a dose of 3 Gy 1, 6, and 24 h after IR refer to Figure S8C. (C–J) Mitochondrial morphology in tumor cancer cell lines 1 h after irradiation with 3 Gy. Representative transmission electron micrographs of A549 (C), NCI-H460 (E), MDA-MB-231 (G), and T98G (I) cell lines and corresponding quantification of the mitochondrial perimeter, area, and mean Feret’s diameter (n = 61–170 mitochondria per condition) at 1 h after IR with a dose of 3 Gy compared with untreated control cells (D, F, H, and J). Scale bar: top row, 1,000 μm; bottom row, 500 μm. p Values calculated using unpaired two-tailed t test; ns, not significant. See also Figure S8.

The similar pattern of the metabolic response of normal and cancer cells to IR described above suggested that general, potentially physical oxidizing effects induced by IR might cause the observed mitochondrial shutdown across cell types. To elucidate if the induction of reactive oxygen species (ROS) as consequence of the IR-induced radiolysis of water might contribute to IR-induced mitochondrial damage, we analyzed if we could attenuate the mitochondrial shutdown by the addition of a chemical ROS scavenger. In fact, pre-treatment of cancer cells with mitochondrial ROS scavenger (MitoTempo [Murphy and Smith, 2007]) in combination with a single dose irradiation reduced ROS formation (Figure S8J) and partially rescued cells from the loss in mitochondrial function (Figures 4A, S8A, and S8B). Many enzymes operating in mitochondria, e.g., in the respiratory chain, are dependent on Fe^2+^ for their function (Levi and Rovida, 2009; Xu et al., 2013). Since IR is well known to oxidize iron ions *ex vivo* (Fregene, 1967), we speculated that exposure to IR alters the oxidative state of iron ions and thereby disturbs cellular iron homeostasis and mitochondrial function. Indeed, exposure to a single-dose irradiation (3 Gy) resulted in increased Fe^3+^/Fe^2+^ ratios (Figures 4B and S8C) at 1 h after irradiation. The basal iron status was reconstituted within 24 h after irradiation as indicated by the reduction in the Fe^3+^/Fe^2+^ ratios (Figures 4B and S8C). A similar time-dependent pattern was observed by using an Fe^2+^-specific fluorescent sensor (RPA (Rhodamine B-[(1,10-phenanthrolin-5-yl)- aminocarbonyl]benzylester)), which is quenched by mitochondrial Fe^2+^ species (Figure S8D). Finally, combining IR with an iron chelator, Deferoxamine (DFO), almost abrogated IR-induced mitochondrial shutdown in all tested cell lines (Figures 4A, S8A, and S8B).

A single high-dose irradiation with 3 Gy induced neither cell death nor apoptosis levels higher than 5% within the first 24 h (Figures S8F–S8I). Moreover, by detailed studies of mitochondrial morphology and mitophagy activity we could exclude the induction of major damage to mitochondrial structures and mitophagy as mechanisms underlying the mitochondrial shutdown observed at 1 h after irradiation (Figures 4C–4J and S8K).

Taken together, our results indicate that IR-induced formation of ROS and massive iron oxidation are causative for the mitochondrial shutdown induced by exposure of cells to a single high dose of irradiation. Moreover, our findings suggest that the importance of compensatory glycolysis to restore mitochondrial function might be linked to the contribution of glycolytic activity to the defense of cells against radiation-induced oxidative stress (Floberg and Schwarz, 2019; Rashmi et al., 2018), potentially also including the homeostasis of Fe^3+^/Fe^2+^ (Li et al., 2019; Oexle et al., 1999).

### Mitochondrial respiration recovery after shutdown and DNA repair is compromised by inhibition of glycolysis

**Figure 5:**
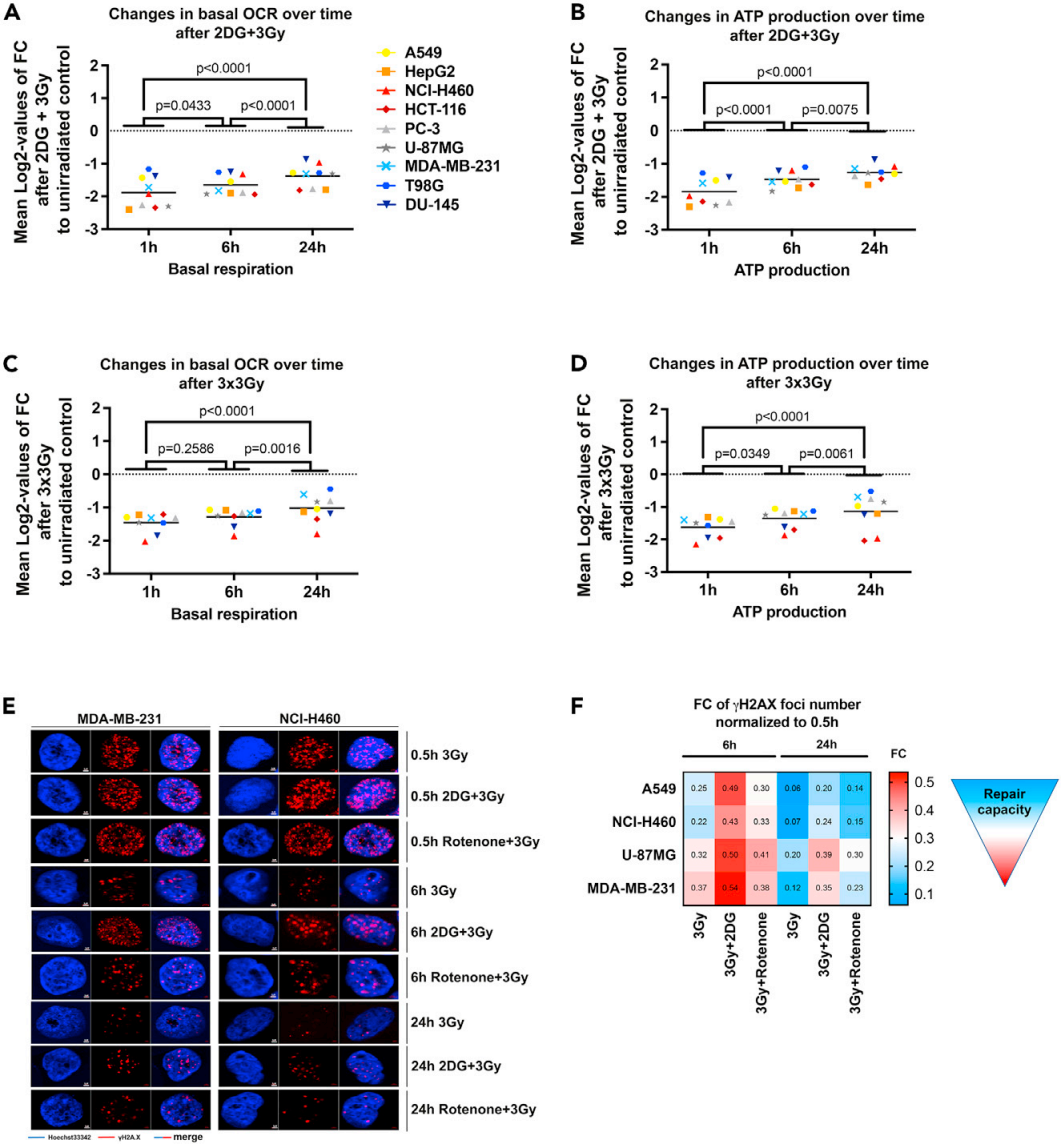
Mitochondrial respiration recovery, DNA repair, and survival after mitochondrial shutdown are compromised by inhibition of glycolysis (A and B) Log2-values of changes to measured basal respiration (A) and ATP production (B) induced by combination treatment with 2DG (20 mM) and IR with a single dose of 3 Gy compared with non-irradiated controls 1, 6, and 24 h after treatment. (C and D) Log2-values of changes to measured basal respiration (C) and ATP production (D) induced by fractionated dose of 3 × 3 Gy applied every 24 h for 3 consecutive days compared with non-irradiated controls 1, 6, and 24 h after the last IR dose. Mean values of n = 8–16 wells per cell line from N = 2 independent experiments are indicated. p values were calculated using two-way ANOVA with Tukey’s multiple comparisons post hoc test. All metabolic parameters were normalized to Hoechst intensity (relative fluorescence units, RFU) in each well. OCR, oxygen consumption rate; FC, fold change. (E) Representative pictures of nuclear γH2A.X on NCI-H60 and MDA-MB-231 cell lines treated with 2DG (20mM) or Rotenone (0.8 μM) in combination with IR (3 Gy) at the indicated time points. Blue, Hoechst 33342; red, γH2A.X. Scale bar: 2 μm. (F) Heatmap representing fold change (FC) values of measured γH2A.X foci number per area normalized to 0.5 h time point after treatment with IR (3 Gy) alone or in combination with 2DG (20 mM) or Rotenone (0.8 μM) at 6 and 24 h. For corresponding DNA repair kinetics determined using the γH2A.X assay with statistical test refer to Figure S9D. See also Figures S8 and S9.

So far, we demonstrated that IR acutely delays and abolishes full recovery of mitochondrial respiration and drives a faster recovery of glycolytic parameters. We thus hypothesized that compensatory glycolysis could ensure energy provision upon IR treatment. In fact, we found prolonged mitochondrial shutdown in irradiated cancer cells pre-treated with the glycolysis inhibitor 2-deoxy-d-glucose (2DG) (Figures 5A, 5B, and S9A) compared with IR alone (Figures 2B, 2C, and S8L).

In line with these findings, inhibition of the DNA repair enzyme poly(ADP-ribose) polymerase 1 (PARP1), a known negative regulator of glycolysis (Fouquerel et al., 2014), by pharmacologic inhibition with the PARP inhibitor PJ34, partially rescued IR-induced drop in glycolytic capacity at 1 h in irradiated cancer cells (Figure S8E).

Clinical radiotherapy uses application of fractionated IR doses. We thus aimed to explore the relevance of the observed effects for clinically more relevant fractionated radiation schedules by assuming repeated cycles of mitochondrial shutdown (Figures 5C, 5D, and S9B). As depicted in Figures 5C and 5D, application of fractionated irradiation with a fractionated dose of 3 × 3 Gy every 24 h lowered all tested parameters (basal and maximal respiration, ATP production) when examined 1 h after the last IR dose. Even more important, the assumed repeated cycles of mitochondrial shutdown by fractionated irradiation with 3 × 3 Gy delayed the mitochondrial recovery (Figures 5C and 5D) compared with a single dose of 3 Gy (Figures 2B, 2C, and S8L).

To gain more insight into the functional relevance of the observed metabolic changes for the assumed link between cancer metabolism and DNA repair kinetics, we analyzed the time-dependent removal of γH2A.X foci after interfering with either compensatory glycolysis by 2DG or with the mitochondrial function by rotenone in four selected cell lines representing different genetic groups defined above (Figures 5E, 5F, and S9D). Rotenone slightly increased the number of residual γH2A.X foci at 24 h after IR, whereas the inhibition of compensatory glycolysis by 2DG efficiently slowed the removal γH2A.X foci at 6 and 24 h after IR, respectively (Figures 5E, 5F, and S9D). These effects were observed in all four cell lines independent of the genetic subgroup. Combining IR with 2DG increased residual γH2A.X foci (Figure 5F) and radiosensitized all tested cell lines, supporting the functional relevance of compensatory glycolysis for the survival of irradiated cancer cells (Figure S8M).

## Discussion

To the best of our knowledge this study provides the first mathematical model of an integrated metabolic IR response of cancer cells with different origins and diverse genetic backgrounds. This model was obtained by the extraction of a general correlation between the IR-induced alterations in mitochondrial metabolic parameters from extracellular flux analyses and the repair kinetics of IR-induced DSBs during the first 24 h after irradiation. Despite the wide heterogeneity of the screened cancer cell lines, we observed a similar pattern of the metabolic response to a single irradiation with 3 Gy regardless of the genetic background. This unified cellular response involved a pronounced and sustained inactivation of mitochondrial respiration, which we termed mitochondrial shutdown, presumably as a consequence of radiation-induced oxidative damage to critical mitochondrial enzymes or their iron-dependent co-factors. The mitochondrial shutdown was paralleled by a rapid downregulation of glycolytic activity. These quick metabolic responses to irradiation were followed by a time-dependent gradual and sequential reconstitution of glycolysis and mitochondrial function. The dynamics of recovery from IR-induced mitochondrial shutdown depended on the genetic background. These novel observations, identified by mathematical modeling, suggest that mechanisms compensating for early mitochondrial shutdown (such as glycolysis) or supporting recovery from mitochondrial shutdown may offer vulnerabilities for a targeted radiosensitization. Further investigations are needed to decipher the potential impact of early mitochondrial shutdown on the long-term survival of irradiated cancer cells. The observation on the faster recovery of irradiated normal epithelial lung cancer cells from IR-induced mitochondrial shutdown compared with cancer cells suggests that targeting metabolic bottlenecks of IR-induced metabolic shutdown may even allow for a cancer cell-specific radiosensitization.

Consistent with our findings, others reported that yeast avoid respiration upon DNA damage, presumably to reduce further harmful effects on DNA (Simpson-Lavy et al., 2015). The IR-induced mitochondrial shutdown observed in our study may thus have similar protective effects in mammalian cells. Furthermore, by decreasing oxygen consumption the IR-induced mitochondrial shutdown may contribute to the early re-oxygenation observed in the microenvironment of irradiated tumors (Crokart et al., 2005; Gallez et al., 2017). Instead, the reduced mitochondrial energy supply may reduce the proliferation rate of cancer cells noticed in irradiated tumors (Bol et al., 2015; Gabry et al., 2018; Lu et al., 2015). Of interest, the IR-induced mitochondrial impairment was followed by a rapid resumption of glycolytic activity during gradual reconstitution of mitochondrial respiration in all cell lines, irrespective of the genetic background or mutational load. We assume that the inversely proportional exploitation of mitochondrial and glycolytic mechanisms is required to maintain energetic and redox homeostasis during recovery of cells upon IR. Glycolysis might thus be a general compensatory mechanism to allow cancer cells to adapt to and survive conditions of endogenous and exogenous oxidative stress (Liemburg-Apers et al., 2015).

In line with our observations, a recent study reported the relevance of glycolysis to provide energy and nucleotides (via the pentose phosphate pathway) during DNA repair, which is initiated directly after exposure to genotoxic agents (Qu et al., 2017). The observed initial decrease of glycolytic activity within the first hour after IR observed in our study might thus reflect an exploitation of basal cellular energetic and metabolic resources to fuel energy and metabolites required for DNA damage response mechanisms and DNA repair early after irradiation when glycolysis and oxidative phosphorylation are impaired. Consistent with our findings, others demonstrated a decrease in glycolytic activity of glioblastoma cancer cells 1 h after treatment with methylnitronitrosoguanidine and linked this decline with hexokinase 1 (HK-1) inhibition by poly(ADP-ribose) polymerase 1 (PARP1)-mediated poly(ADP-ribose) (PAR) synthesis (Fouquerel et al., 2014). IR-induced PARP1 activation is also an essential step for the activation of DNA repair pathways (Homologous Recombination Repair (HRR), base excision repair, and alternative end joining) (Fouquerel et al., 2014; Qu et al., 2017). In our hands, the glycolytic capacity of irradiated cancer cells was higher when cells were treated with the PARP inhibitor PJ34 compared with cells receiving irradiation only, corroborating a role of PARP1 activity in the rapid decline of glycolytic activity in irradiated cancer cell line panel.

Yet, further investigations are needed to reveal whether PARP-1-dependent effects on glycolytic activity of irradiated cancer cells are also due to inhibition of HK-1.

We speculate that the IR-induced mitochondrial shutdown will force cells to exploit alternative energy sources to support repair of IR-induced DNA damage, particularly DNA DSB repair, which is considered a highly energy-consuming process (Bakkenist and Kastan, 2004; Hopfner et al., 2004; Paull and Gellert, 1999; Qin et al., 2015). Herein, rapid resumption of compensatory glycolysis will allow cancer cells to fuel highly energy-demanding DNA repair processes despite temporary loss of mitochondrial function upon depletion of basal cellular energetic and metabolic resources. This assumption is based on our observation that pharmacologic inhibition of glycolysis in irradiated cancer cells by 2DG not only impaired the onset of compensatory glycolysis but also significantly delayed both recovery of irradiated cancer cells from mitochondrial shutdown and the resolution of γH2A.X foci.

In line with our findings, previous reports observed potent radiosensitizing effects of 2DG in pancreatic cancer, glioblastoma, and radioresistant cervical cancer cells (Coleman et al., 2008; Mohanti et al., 1996; Prasanna et al., 2009). Furthermore, inhibition of oxidative phosphorylation with Rotenone also only affected the resolution of γH2A.X at later time points (6–24 h), suggesting that mitochondrial respiratory chain function will have to recover from radiation-induced damage to resume function and support DNA repair with energy and essential metabolites (Chandel, 2015; Lu et al., 2015; Qin et al., 2015; Weinberg and Chandel, 2015). Clearly, irradiated cancer cells can benefit from ATP supplied by a dynamic enhancement in mitochondrial oxidative phosphorylation (Lu et al., 2015; Qin et al., 2015). For example, cell lines with defects in homologous recombination DSB repair pathway increase the activity of mitochondrial respiration to provide NAD^+^ and ATP for poly(ADP-ribose) polymerase (PARP)-dependent DNA repair (Lahiguera et al., 2020). Herein, recently identified mechanisms of mitochondrial-nuclear signaling have been linked to retrograde pro-survival response, pointing to a two-way interconnection of mitochondrial metabolism and DNA repair (Desai et al., 2020; Wu et al., 2019).

Using cancer cell lines from different entities with various genetic backgrounds, we were able to reveal a potential correlation of DNA repair kinetics with the genetic background (mutations in Ras and p53), the mitochondrial shutdown, and compensatory glycolysis. Although we found that activation of PARP1 contributes to the rapid decline of glycolytic activity in irradiated cancer cells, presumably as a consequence of IR-induced DNA damage, the mechanisms underlying the mitochondrial shutdown remained elusive. By using flow cytometry as well as biochemical and microscopy methods including TEM, we could exclude that those structural mitochondrial changes contribute to impaired mitochondrial function at the time of the observed IR-induced mitochondrial shutdown that was most pronounced at 1 h after irradiation. Furthermore, the unified reduction of mitochondrial respiration observed across all tested cell lines argued against a predominant role of oncogenic signaling for this effect. Instead, the similar pattern of the metabolic response of normal and cancer cells to IR strongly suggested that the mitochondrial shutdown might rather be a consequence of the physical effects induced by IR resulting in secondary changes in cellular bioenergetics, rather than altered activation of metabolic enzymes AMPK, SIRT3, or PARP1 described in response to IR (Fouquerel et al., 2014; Liu et al., 2015; Sanli et al., 2010). This prompted us to investigate if physical effects induced by IR, e.g., oxidation of cellular components, might cause the observed IR-induced mitochondrial shutdown across cell types. These investigations revealed that the addition of a mitochondrial ROS scavenger partially rescued cells from the loss in mitochondrial function, pointing to the contribution of radiation-induced mitochondrial oxidative stress to the decline in mitochondrial function. Furthermore, we observed an increase in the oxidative state of iron (increased Fe^3+^/Fe^2+^ ratio) at the time of the IR-induced mitochondrial shutdown and were able to rescue cells from the IR-induced mitochondrial shutdown by the iron scavenger DFO. IR acts as a strong oxidant, including also the oxidation of iron ions (Fregene, 1967), and mitochondria are major hubs of iron accumulation and utilization (Paul et al., 2017): for example, iron is needed as a cofactor in heme- or iron-sulfur cluster-containing proteins functioning in the mitochondrial respiratory chain; furthermore, various mitochondrial mono- or dioxygenases use iron ions as co-factors. Thus, our observations strongly suggest that IR-induced physical events, particularly the oxidation of iron ions, are major contributors to the radiation-induced mitochondrial shutdown and metabolic dysfunction. Such physical effects on iron ion oxidation state would also provide an explanation for our findings that the subsequent recovery of mitochondrial activity was delayed and incomplete when applying a fractionated dose of 3 × 3 Gy. In fact, the suggested role of physical events in radiation-induced metabolic dysfunction implicates that fractionated irradiation will cause a more pronounced and long-lasting metabolic dysfunction.

Yet further studies are needed to gain a better mechanistic understanding of the radiation mitochondrial shutdown and also to address potential differences in the importance of cell metabolism upon hypo-fractionated versus hyper-fractionated radiation schedules, or when using different dose rates or radiation qualities (e.g., particle versus photon irradiation) in the future.

Taken together, targeting the recovery of mitochondrial respiration or compensatory mechanisms of energy and metabolite supply is suited to compromise DNA repair of irradiated cancer cells. Even more important, statistical modeling of our DNA repair and metabolic data demonstrated a general correlation between IR-induced alterations in mitochondrial metabolic parameters detected in the extracellular flux measurements and the kinetics of IR-induced DNA DSB repair. This analysis underlines the reliance of IR-induced DNA repair on the cellular ability to compensate the energetic catastrophe evoked by the mitochondrial shutdown. Although mitochondrial shutdown was also observed in normal lung epithelial cells, the recovery process of mitochondrial function to normal levels was faster in normal cells compared with cancer cells. This observation corroborates finding from demonstrating significant differences in the metabolic plasticity between tumor and normal tissue (Budczies et al., 2012; Reznik et al., 2018) pointing to various means to recover from the IR-induced mitochondrial shutdown. It thus seems plausible that non-proliferating normal tissue cells use distinct metabolic pathways to recover from an IR-induced mitochondrial shutdown than oncogene-driven proliferative cancer cells creating oncogene-driven metabolic bottlenecks for survival of cancer cells, particularly during therapy-induced stress.

The capability of cancer cells to recover from IR-induced mitochondrial damage was determined by their metabolic plasticity, e.g., their ability to rapidly activate the glycolytic pathway, presumably to provide energy and nucleotides for the repair of DNA DSBs. Notably, the metabolic flexibility to recover from the IR-induced mitochondrial shutdown depended on the genetic background. Using isogenic cells lines we could demonstrate that the introduction of an oncogenic mutation in KRAS rendered NCI-H838 cancer cells more capable of recovering from IR-induced mitochondrial shutdown and this was associated with an improved ability to remove IR-induced DNA damage foci. By contrast, the deficiency of p53 in HCT-116 cells attenuated the resumption of mitochondrial activity upon irradiation and this went along with slowed DNA repair kinetics. We thus conclude that the energy supply and demand in irradiated cancer cells are orchestrated by mutations in several cancer-promoting genes, e.g., *RAS* and *TP53*, which are also linked, among others, to DNA repair capacity (Hähnel et al., 2014; Krause et al., 2016; Liu et al., 2015; Toulany et al., 2016) and survival (Viale et al., 2014).

Radiotherapy is applied locally to the tumor with high precision and thus provides the opportunity to unmask metabolic vulnerabilities specifically in the irradiated cancer cells. Herein, the IR-induced early mitochondrial shutdown creates a common metabolic vulnerability in the form of compensatory glycolysis across cell lines.

Yet distinct pathways will control activation of compensatory glycolysis and recovery of oxidative phosphorylation upon irradiation in cancer cells with distinct genetic backgrounds so that individual metabolic treatments may be required for radiosensitization. Pharmacological inhibition of the underlying metabolic dependencies and compensatory metabolic pathways will allow enhancement of cytotoxic efficacy of IR in cancer cells. Furthermore, our screen of the cancer cell line panel suggests a strong correlation between cancer metabolism and DNA repair within the first 24 h after IR. Yet further mechanistic studies are required to dissect the molecular mechanisms linking the mitochondrial shutdown to suggested defects in DSB repair and to define common and individual pathways driving compensatory glycolysis and recovery of mitochondrial respiration to fuel the repair of radiation-induced DNA lesions.

In conclusion, combining systematic radiobiological investigations with mathematical modeling of the obtained results represents an innovative approach for the discovery of therapeutic targets. Our study gained first insight into a potential role of the genetic background for the ability of the cancer cells to activate compensatory glycolysis and resume mitochondrial function upon irradiation. Future studies will uncover the importance of the genetic background for the ability of cancer cells to cope with radiation-induced damage by rewiring their metabolism and help to generate new hypotheses on general and individual mechanisms of radiosensitivity.

### Limitations of the study

Our study aimed to reveal potential metabolic bottlenecks of the DNA repair capability by using extracellular flux analysis, DNA repair kinetics, and mathematical modeling. Our screen of the cancer cell line panel suggests a strong correlation between cancer metabolism and DNA repair within the first 24 h after IR. Yet further mechanistic studies are required to dissect the molecular mechanisms linking the mitochondrial shutdown to suggested defects in DSB repair and to define common and individual pathways driving compensatory glycolysis and recovery of mitochondrial respiration to fuel the repair of radiation-induced DNA lesions. Our experimental approach does not allow exploration of the link between a dependency of the cancer cells on basal mitochondrial respiration for survival upon radiotherapy and radiosensitivity. We suspect that additional differences in the genetic background of the cells interact with and orchestrate the metabolic phenotype of the cancer cells and the ability to adapt their metabolism in response to irradiation to fuel DSB repair and cell survival. Herein, glycolysis appears to play an important and central role including also other processes, such as antioxidant defense.

## STAR★Methods

### Key resources table

**Table:** 

REAGENT or RESOURCE	SOURCE	IDENTIFIER
**Antibodies**		

mouse anti-actin HRP-conjugated antibody	Santa Cruz	sc-8432-HRP; RRID: AB_626630
mouse anti-ATP Synthase antibody, (clone 4.3E8.D)	Millipore	MAB3494; RRID: AB_177597
mouse anti-HSP 60 antibody (LK1)	Santa Cruz	sc-59567; RRID: AB_783870
mouse anti-prohibitin 2 antibody (A-2)	Santa Cruz	sc-133094; RRID: AB_2164785
mouse anti-TOMM20 (F-10)	Santa Cruz	sc-17764; RRID: AB_628381
mouse anti-γ-H2A.X Alexa Fluor 647-conjugated (pS139), clone N1-431	BD Bioscience	Cat# 560447; RRID: AB_1645414
anti-mouse IgG-HRP secondary antibody	Cell Signaling	7076; RRID: AB_330924
anti-Rabbit IgG-HRP secondary antibody	Cell Signaling	7074; RRID: AB_2099233
goat-anti-mouse Alexa Fluor 488 secondary antibody	Invitrogen	A-11001; RRID: AB_2534069

**Chemicals, peptides, and recombinant proteins**		

1,2-Propylen oxide	VWR	Cat# 27165.295
2-Deoxy-D-glucose	Sigma-Aldrich	Cat# D8375; CAS: 154-17-6
Agar-Agar	Merck-Millipore	1.01614
Dako Fluorescence Mounting Medium	Dako North America Inc.	S3023
Dalton solution:		
Osmium Tetraoxide	Electron Microscopy Science	Cat# 19134; CAS: 20816-12-0
Potassium dichromate	Carl Roth	Cat# 7953; CAS: 7778-50-9
Deferoxamine (DFO)	Sigma Aldrich	D9533; CAS: 70-51-9
Dihydroethidium (DHE)	Molecular Probes/Invitrogen	D1168; CAS: 104821-25-2
Dulbecco’s Modified Eagle’s (DMEM) medium	Thermo Fisher Scientific	41965039
Epon mixture:		
Glycid Ether (EPON812)	SERVA	Cat# SERVA21045.02; CAS: 90529-77-4
Methylnadic anhydride	SERVA	Cat# SERVA29452.02: CAS: 25134-21-8
2-Dodecenylsuccinic acid anhydride	SERVA	Cat# SERVA20755.0; CAS: 19780-11-1
2,4,6-Tris(dimethylaminomethyl)phenol	SERVA	Cat# SERVA36975.01; CAS: 90-72-2
Ethanol, p.a.	VWR	Cat# 20821.32; CAS: 64-17-5
Fetal Bovine Serum (FBS)	Biochrom	S 0115
Glutaraldehyde solution	Sigma Aldrich	Cat# 49629; CAS: 111-30-8
Hoechst 33342, trihydrochloride, trihydrate	Thermo Fisher Scientific	Cat# H1399; CAS: 875756-97-1
D-(+)-Glucose	Sigma-Aldrich	Cat# G7021; CAS: 50-99-7
L-Glutamine	Thermo Fisher Scientific	Cat# 25030024; CAS: 56-85-9
Lead citrate trihydrate	Fluka Chemika	Cat# 15326; CAS: 6107-83-1
MitoTempo	Cayman Cheimcals	Cat# 16621; CAS: 1334850-99-5
Sodium citrate monobasic	Sigma-Aldrich	Cat# 71497-250G; CAS: 18996-35-5
Sodium pyruvate	Sigma-Aldrich	Cat# P5280; CAS: 113-24-6
Seahorse XF base medium (DMEM)	Agilent Technologies	103334-100
Paraformaldehyd	Carl Roth	Cat# 0335.2; CAS: 30525-89-4
Penicillin-Streptomycin (10,000 U/mL)	Thermo Fisher Scientific	15140122
Phosphate-Buffered Saline (PBS) 1x	Thermo Fisher Scientific	10010023
PJ34	Selleckchem	S7300
Propidium iodide	Sigma-Aldrich	Cat# 81845; CAS: 25535-16-4
Rhodamine B-[(1,10-phenanthroline-5-yl)-aminocarbonyl]benzyl ester (RPA)	Squarix Biotechnology	Cat# ME043; CAS: 408356-71-8
Rotenone	Sigma-Aldrich	Cat# R8875; CAS: 83-79-4
Triton X-100	Carl Roth	Cat# 3051.4; CAS: 9002-93-1

**Critical commercial assays**		

GenePrint 10 System	Promega	B9510
Iron Assay Kit (Colorimetric)	Abcam	ab83366
Seahorse XF Cell Mito Stress Test kit	Agilent Technologies	103015-100
Seahorse XF Glycolysis Stress Test Kit	Agilent Technologies	103020-100

**Experimental models: Cell lines**		

A549	ATCC	CCL185
HepG2	ATCC	HB-8065
DU-145	ATCC	HTB-81
HCT-116	ATCC	CCL-247
HPAC	ATCC	CRL-2119
HSAEC1-KT	ATCC	CRL-4050
MDA-MB-231	ATCC	HTB-26
NCI-H460	ATCC	HTB-177
NCI-H838	ATCC	CRL-5844
NCI-H838 KRAS^G12D^	Horizon Discovery	As a gift
PC-3	ATCC	CRL-1435
PaTuS	DSMZ	ACC-204
T98G	ATCC	CRL-1690
U-87 MG	ATCC	HTB-14

**Software and algorithms**		

Prism 7	GraphPad	https://www.graphpad.com/scientific-software/prism/
ImageJ software	NIH, open source	https://imagej.nih.gov/ij/
FlowJo software	FlowJo, LLC,	https://www.flowjo.com/solutions/flowjo/downloads
Focinator version 2-22 software	(Oeck et al., 2017)	http://focinator.oeck.de/
R software	R Core Team, open source	https://www.r-project.org/
RStudio Open-Source Edition software	RStudio, open source	https://www.rstudio.com/products/rstudio/
R package rstanarm version 2.18.2	(Goodrich et al., 2018)	http://packages.renjin.org/package/org.renjin.cran/rstanarm/2.18.2
shinystan version 2.5.0	(Gabry et al., 2018)	https://mc-stan.org/shinystan/
Stan	(Carpenter et al., 2017)	https://www.jstatsoft.org/article/view/v076i01
Wave 2.4 Desktop software	Agilent Technologies	https://www.agilent.com/en/products/cell-analysis/software-download-for-wave-desktop
Zeiss ZEN pro software	Carl Zeiss	https://www.zeiss.com/microscopy/int/products/microscope-software/zen.html

**Other**		

Cell lines genomic data and analysis (accessed: 2019/09/20)	COSMIC Cell Line Project	https://cancer.sanger.ac.uk/cosmic
EM grids: formvar and carbon coated	Plano	S162-5

### Resource availability

#### Lead contact

Further information and requests for resources and reagents should be directed to and will be fulfilled by the lead contact, Johann Matschke (Johann.Matschke@uk-essen.de), following an approved Material Transfer Agreement between the Institute of Cell Biology (Cancer Research), University of Duisburg-Essen and the receiving institution.

#### Material availability

This study did not generate new unique materials or reagents.

### Experimental model and subject details

#### Cell lines

Cancer cell lines used in this study were chosen to represent a broad heterogeneity of malignant disease in respect to site of origin, cancer type, and oncogenic drivers (especially p53 tumor suppressor and Ras oncogene). A549 (human, male, lung adenocarcinoma), HepG2 (human, male, hepatocellular carcinoma), NCI-H460 (human, male, lung adenocarcinoma), HCT-116 (human, male, colon adenocarcinoma), PC-3 (human, male, prostate adenocarcinoma), U-87 MG (human, male, glioblastoma), MDA-MB-231 (human, female, breast adenocarcinoma), T98G (human, male, glioblastoma multiforme), DU-145 (human, male, prostate adenocarcinoma), HSAEC1-KT (human, male, normal lung epithelial) cell lines were purchased from the American Type Culture Collection (ATCC, Manassas, VA, USA). NCI-H838 (human, male, lung adenocarcinoma) KRAS^WT^ and KRAS^G12D^ were obtained from Horizon Discovery as a gift. All cell lines, except of HSAEC1-KT, were cultured in Dulbecco’s modified Eagle’s medium (DMEM, Life Technologies, Carlsbad, CA, USA) supplemented with 10% fetal bovine serum (FBS) with addition of 1% penicillin-streptomycin (Pen-Strep, Life Technologies) and maintained in a humidified incubator at 37°C and atmosphere containing 5% CO_2_. HSAEC1-KT cells were cultured in Airway Epithelial Media (PromoCell, Heidelberg, Germany). All cell lines were authenticated through STR sequencing and analysis performed with the GenePrint 10 System (Promega Corp., Madison, WI, USA) in accordance with the ANSI/ATCC ASN-0002-2011 standardization of STR profiling guidelines. All cell lines were routinely tested for mycoplasma contamination.

#### Extracellular flux assay – Mito stress test

Cells were plated in XF96 micro-plates (Agilent Technologies, Santa Clara, CA, USA; for seeding densities see Table S1) in DMEM Medium supplemented with 10% FBS and 1% Pen-Strep at least 24h prior to the assay and cultivated in humified incubator at 37°C with 5% CO2. Treatment with MitoTempo (1nM), Deferoxamine (DFO, 50μM) or D-2DG (20mM) was performed 2h before irradiation with 3Gy. For the treatment with a fractionated dose, cells were irradiated 3 times with a dose of 3Gy (3 × 3Gy) every 24h. After irradiation, and 1h prior to the assay designed time point, medium was exchanged to the Seahorse XF Base Medium (Agilent Technologies) with 1-mM sodium pyruvate, 2mM L-glutamine and 10mM D-(+)-glucose (all Sigma-Aldrich, St. Louis, MO, USA), pH 7.4 and incubated at 37°C without CO_2_. During assays, oxygen consumption rate (OCR) and extracellular acidification rate (ECAR) were measured in parallel using a Seahorse XFe 96 analyzer (Agilent Technologies). Seahorse XF Cell Mito Stress Test kit (Agilent Technologies) containing injections of oligomycin, carbonyl cyanide-4-(trifluoromethoxy) phenylhydrazone (FCCP), followed by combined injection of rotenone and antimycin A was performed according to manufacturer’s protocol (see Table S1 for detailed information). In brief, after 3 measurements of basal parameters, injection of oligomycin, FCCP and rotenone/antimycin A were performed, separated by each time point by 3 measurements of OCR/ECAR parameters. For individual well normalization of cell number, DNA content fluorescence was measured after cells were fixed with 4% PFA (Carl Roth, Karlsruhe, Germany) and stained with 10-μg/mL Hoechst 33342 (Sigma-Aldrich) solution after each assay. Data were analyzed using Wave 2.4 software (Agilent Technologies). For Figures 1A and 2A–2D mean values of n = 12–16 wells per cell line from N = 2 independent experiments are indicated. All metabolic parameters were normalized to Hoechst intensity (relative fluorescence units, RFU) in each well.

#### Extracellular flux assay – glycolysis stress test

Treatment with PJ34 (4μM) was performed 2h before irradiation with 3Gy. After irradiation, exactly 1h before the designated time point, cells were washed with 1x PBS and incubated in unbuffered DMEM (containing additionally 1 mM L-glutamine), pH 7.4, at 37°C in a non-CO2 incubator. Subsequently, three extracellular acidification rate measurements were performed using the Seahorse analyzer, and measurements were repeated following injection of glucose (10 mM), oligomycin (1μM) and 2-deoxy-glucose (50mM) (Agilent Technologies). Extracellular acidification rates (glycolytic flux parameters) were determined according to the manufacture’s protocol and calculated as shown in Table S1. Normalization to the cell number was performed as described above and was followed by data evaluation using Wave 2.4 software.

#### Irradiation

Cells were exposed to 3Gy of x-ray ionizing radiation using photon beams produced with a X-RAD 320 X-Ray Biological Irradiator with a MIR-324 X-ray tube, with 1.65mm beam conditioning filter (Precision X-Ray, North Branford, CT, USA). Irradiations, with an initial energy of 320 keV and a rate of 3.75Gy/min at a distance of 50cm from the x-ray tube window were controlled by a parallel dosimetry with a PTW 7862 parallel plate transmission chamber and PTW UNIDOS dosimeter (Precision X-Ray).

#### Electron microscopic study of mitochondrial morphology after irradiation

A549, NCI-H460, MDA-MB-231 and T98G cells were plated in T25 cell culture flasks in DMEM Medium supplemented with 10% FBS and 1% Pen-Strep and cultivated in humified incubator at 37°C with 5% CO2 until 100% confluence. Upon irradiation with 3Gy, cells were fixated at the indicated time points (1h and 24h after irradiation) with 2.5% glutaraldehyde in PB for 48h. Control cells without irradiation treatment were fixed together with the cells of the 24h post irradiation time point. After cells were washed with PB, they were detached from the flasks bottom with cell scrapers and centrifuged at 5 000 rpm for 3min. Afterwards, cell bands were embedded in Agar-Agar (4% in PB). Cell blocks were incubated with Dalton solution for 90min and washed five times with PB for 5min. Next, the specimens were dehydrated through an ascending ethanol series as described before (Krause et al., 2016). After incubation in propylene oxide for 20min, an ascending series of Epon mixture in propylene oxide was applied, first 3:1 for 1h, then 1:1 for 1h, followed by 1:3 for 2h, and finally, specimen blocks were penetrated by pure Epon over night at 20°C. After replacing fresh Epon, polymerization was performed at 60°C for 48h. 50nm sections were cut with an Ultracut E Reichert-Jung (Leica Microsystems GmbH, Wetzlar, Germany) collected on Formvar-coated grids and contrasted with lead citrate for 5min. Samples were analyzed with a Zeiss LEO 910 transmission electron microscope equipped with a digital CCD camera. ImageJ 1.51s (National Institutes of Health, Bethesda, MD, USA) was used for the evaluation of single mitochondrial morphological parameter, like area, perimeter, and mean Ferret’s diameter.

#### Mitophagy assays

##### Western blotting

Cells were plated 24h prior to radiation treatment. Cells were washed in ice-cold PBS at the indicated time points and lysed in lysis buffer (Tris-HCl, pH 7.4, 50 mM; NaCl, 250 mM; Igepal 0.5%) containing proteases (Complete Protease Inhibitor Cocktail, Roche) and phosphatase (PhosSTOP Phosphatase Inhibitor Cocktail, Roche) inhibitors for 10min at 4C. Lysates were cleared by centrifugation at 16,000 g for 10 minutes at 4°C, mixed with 4x Loading buffer and boiled 10min at 95°C. Proteins were analyzed by western-blotting as in Vega-Rubin-de-Celis et al. (2010). Upon resolving in SDS-PAGE and transfer to PVDF membranes (Bio-Rad) samples were blocked in 5% non-fat dry milk in TBS-T (TBS containing 0.1% Tween-20) for 1h at room temperature. Membranes were incubated with primary antibodies diluted in 3% BSA-TBS-T overnight at 4°C. After washing three times with TBS-T, membranes were incubated with HRP-conjugated secondary antibodies [Cell Signaling, HRP-linked anti-mouse (cat. number #7076) or anti-rabbit (cat. number #7074) in 5% milk-TBS-T for 45 minutes at room temperature.

Primary antibodies were from Santa Cruz: TOMM20 (sc-17764, 1:500), PHB2 (sc-133094, 1:500), HSP60 (sc-59567, 1:500), Actin-HRP (sc-8432-HRP, 1:5000).

##### Immunofluorescence

Cells were seeded on 24-well imaging plates (Eppendorff, Hamburg, Germany) and radiated 24h later. Cells were fixed at the indicated time points in 2% PFA in PBS for 10 minutes at room temperature, followed by three 5 minutes washes with PBS. Samples were permeabilized in 0.5% Triton-X100 in PBS for 2 min 30 seconds and rinsed in PBS. Blocking was performed in 0.5% BSA-PBS for 30 minutes. ATP5B antibody (Millipore, MAB3494) was diluted to 1:500 in 0.5% BSA-PBS and incubated overnight at 4C. Alexa Fluor 488-conjugated secondary antibody (1:1000; Invitrogen) was incubated for 1 hour at room temperature in 0.5% BSA-PBS containing Hoechst. ATP5B dots were counted in at least 50 cells per condition for quantification under a Leica DMi8 microscope.

#### Detection of iron status (Iron Assay kit, Abcam)

Cells were plated, irradiated on the following day with a dose of 3Gy and lysed 1, 6 and 24h after irradiation according to manufacturer’s protocol (Abcam Iron Assay Kit ab83366, Abcam, Cambridge, UK). In brief, Fe^2+^ reacts with Ferene S to produce a colored complex with absorbance at 593nm. Fe^3+^ can then be reduced to Fe^2+^, thereby enabling to measure total iron (Yang et al., 2020; Zhou et al., 2020).

#### Detection of Fe^2+^ by RPA using flow cytometry

Cells were plated in a 6-well plate and irradiated on the following day with a dose of 3Gy. Cell pellets were stained with 40μM RPA (Squarix Biotechnology, Marl, Germany) for 15min and mean fluorescence intensity (MFI) was detected by flow cytometry (BD Accuri C6, Becton Dickinson, Heidelberg, Germany; FL-2). Fold changes were quantified to the corresponding non-irradiated controls. In brief, RPA fluorescence is quenched by Fe^2+^ allowing the determination of iron reduction (Petrat et al., 2002; Santambrogio et al., 2015).

#### Determination of reactive oxygen species (ROS)

To quantify cellular ROS production, cells were stained for 30 min at 37°C with 5 μM of Dihydroethidium (DHE) (Molecular Probes/Invitrogen, Carlsbad, CA, USA). DHE-positive cells were detected by flow cytometry (BD Accuri C6, Becton Dickinson, Heidelberg, Germany; FL-2). Fractions of gated positive cells (at least 10,000 events) with higher fluorescence were evaluated. Fold changes were quantified to the corresponding non-irradiated controls.

#### Short-term cell death, apoptosis, and cell cycle assays

The fraction of dead cells was quantified by flow cytometry (FACS Calibur, Becton Dickinson Heidelberg, Germany; FL-2) of PI–stained cells. Cells were incubated for 30min in the dark with PI (10μg/ml) in PBS and measured within 1 hour. Fractions of gated positive cells (at least 10,000 events) with higher fluorescence were evaluated. For quantification of apoptotic DNA-fragmentation (sub-G1 population) or Cell cycle of gated positive cells (at least 10.000), cells were incubated for 30min in the dark with a staining solution containing 50μg/ml PI in a hypotonic citrate buffer 0.1% sodium citrate and 0.05% Triton X-100 and subsequently analyzed by flow cytometry (BD FACS Calibur, Becton Dickinson, Heidelberg, Germany; FL-2) (Nicoletti et al., 1991).

#### Colony formation assays

Clonogenic cell survival in response to the respective treatments was determined comparing the clonogenic survival of cells cultured under standard culturing conditions (20% O_2_, 5% CO_2_, 37°C). For treatment, exponentially growing cells were seeded in tissue culture flasks, incubated under standard culturing conditions (20% O_2_, 5% CO_2_, 37°C) and irradiated 24h later (0 to 8Gy) without or with prior D-2DG (20 mM) treatment. D-2DG treatment was performed 2 h prior to irradiation. After completion of the treatments, cells were incubated for 24h, washed, collected (0.05% Trypsin, 0.01 EDTA), and plated to 6 well plates at densities of 200 to 3200 cells per well (delayed plating). The cell viability was checked before plating the cells by using CASY COUNT (Omni Life Science, Bremen, Germany) and only the number of viable cells were used for the calculation of necessary cell numbers for plating. Plates were subsequently incubated for 9 days under standard conditions before quantification of colony formation. For this, cells were fixed in 3.7% formaldehyde and 70% ethanol, stained with 0.05% Coomassie blue, and colonies of at least 50 cells were counted by GelCount (Oxford Optronix, Oxfordshire, UK). The plating efficiency and surviving fraction (SF) were calculated as described elsewhere (Franken et al., 2006).

#### Immunofluorescence staining

Cells were pretreated with D-2DG (20mM) or Rotenone (0.8μM) for 2h before irradiation with 3Gy. At a given time after IR (30min, 2h, 6h and 24h) cells were fixed and permeabilized with 3% para-formaldehyde and 0.2% Triton X-100 (Carl Roth) in PBS for 15min at room temperature. After washing with PBS, cells were blocked overnight with 2% normal goat serum in PBS. Incubation with anti-γ-H2A.X Alexa Fluor 647-conjugated antibody (AF-647, Catalog No. 56044, BD Bioscience, Franklin Lakes, NJ, USA) was performed for 1h at a 1:100 dilution in blocking buffer. Samples were washed three times with PBS followed by staining for 15min in the dark with 0.2% (w/v) Hoechst33342 in PBS. Samples were again washed three times with PBS, mounted with the DAKO mounting medium (DAKO, Glostoup, Denmark) and stored at 4°C in the dark.

#### Fluorescence images acquisition

Single layer fluorescence images were taken with a Zeiss AxioCam MRm (1,3883 x 1,040 pixels) at a Zeiss Axio Observer Z1 fluorescence microscope with Plan-Apochromat 63x/1.40 Oil M27 lens, 49 DAPI filter (Hoechst33342 detection), 78 HE ms CFP/YFP filter (γ-H2.AX AF-647 detection) and a transmission grid VH ‘ApoTome’ (Carl Zeiss, Oberkochen, Germany). Images were taken with three fourth of the maximum intensity without overexposure. The pictures were saved as 16-bit multi-channel Carl Zeiss Image files with no further editing. At least 80 nuclei per cell line per experiment were analyzed by counting γH2A.X foci. N = 3 independent experiments were performed and used for the analysis.

#### Foci quantification and analysis

The ImageJ macro Focinator version 2.2 and the R-based batch mode were applied to automatically count γH2.AX foci as previously described (Oeck et al., 2015, 2017). The Focinator counts foci for each pre-selected region of interest (ROI) defined as nuclei by Hoechest33342 staining automatically. Foci are detected as a command of Image ‘Find maxima’ and discriminated from the surrounding pixels out of 16-bit grey scale image by signal peaks coming from fluorescence intensities. The same values of background correction and cut-off were applied to all analyzed pictures within all experiments. Obtained foci counts were normalized to the respective area (Pixel^2^) of the ROI. The software, instructions and supporting information are obtainable at http://www.focinator.oeck.de.

### Quantification and statistical analysis

#### Statistical analysis

Classical statistical analyses were completed using two-way ANOVA with Tukey's multiple comparisons post hoc test, two-way ANOVA with Šídák's multiple comparisons post hoc test, or unpaired two-tailed t-test. Analyses were performed using GraphPad Prism version 7 (GraphPad Software, Inc., San Diego, CA). For each analysis p-values were either reported as numbers or represented by stars, where: ∗ - p ≤ 0.05, ∗∗ - p ≤ 0.01, ∗∗∗ - p ≤ 0.001, ∗∗∗∗ - p ≤ 0.001, and ns – not significant.

Log-transformed measurements of basal respiration, ATP production, maximal respiration, glycolytic capacity, at time 0h (Figures 1B–1D and 1F) were modeled with multilevel generalized linear models (GLMs) assuming normal distributions at all levels. We inferred an overall mean of the log-values, and also deflections from that mean as function of factor cell line and replicate, with the latter nested into the former. The distributions of the GLM parameters were inferred by Bayesian computation with Stan (Carpenter et al., 2017) as available through the stan_lmer function of R-package rstanarm, version 2.18.2 (Goodrich et al., 2018) with weakly informative default priors. The quality of model fits was inspected by Posterior Predictive Checks with the shinystan software (Gabry et al., 2018). Distributions of the cell line specific intercepts of metabolic parameters were summarized graphically by 95% and 50% highest density intervals (HDIs) of probability, and numerically by accumulated probabilities *P*- (“P minus”) of negative effects. Time courses of glycolytic capacity for all cell lines (Figure 2F) were modelled as a single multilevel GLM, analogous to Figure 1F, but with additional factor time.

#### Statistical model of γ-H2A.X foci dynamics

A multilevel statistical model was developed and applied to summarize the dynamics of γH2AX foci over time (for formulas see supplemental information). For each replicate *r* and time point *t,* a binomial distribution was assumed for the number of foci. The γH2.AX foci probability per unit area, *p*_*r,t*_ (= success probability), of that binomial distribution was drawn from a beta distribution with time dependent parameters. The size of the unit area was 0.1∗Pixel^2^; with this unit area, the value of the probability p_r,t_ was safely lying between 0 and 1. Above the replicate level, we had another, replicates-overarching beta distribution with time dependent success probability *p*_*t*_. The variance of that latter beta distribution was modeled as coming from a χ^2^ distribution with *k*_*t*_ degrees of freedom. Priors for the highest levels were all χ^2^ distributions with degrees of freedom parameter set to 2. For each cell line, a separate model of this type was fitted. The model was implemented in Stan (Carpenter et al., 2017), and model parameters with credible intervals of marginal distributions inferred by Bayesian analysis. During the development process several alternative models were validated by posterior predictive checks (Figure S4), i.e., comparisons of models and reality. Pairs of alternative models were compared with respect to predictive performance by approximate leave-one-out cross-validation (Vehtari et al., 2017). The courses of γ-H2.AX foci dynamics were compared between cell lines and plotted in a heatmap (Figure 3E). To this end we collected the five values of *p*_*t*_ for times *t* = 0, 0.5, 2, 6, 24 *h* for each cell line *i* in a five-dimensional vector q_*i*_. Then we evaluated for each pair of cell lines *i*, *j* the Euclidean distance d (q_*i*_, q_*j*_), and plotted all pairwise distances as colors in a heatmap.

#### Linear models

For the linear models in Figure 3 we first interpolated γH2.AX foci numbers per unit area (0.1∗Pixel^2^) from those at 0.5 h and 2 h to have γH2.AX foci numbers per unit area and metabolic measurements at the same four time points 0h, 1h, 6h, and 2 4h (results in Figure S6A). For each pair of cell lines we then took γH2.AX foci probabilities per unit area and log- metabolic parameter at these four time points and inferred probability distributions of slope (Figure S7A) and intercept (Figure S7B) of a linear model, relating γ-H2.AX foci probabilities per unit area to log-metabolic parameters. Inference was performed by Bayesian analysis with R package rstanarm (Goodrich et al., 2018). The linear models in Figure 3 are represented by least square fits.

## Data Availability

This study did not use any unpublished custom code, software, or algorithm. Any additional information required to reanalyze the data reported in this paper is available from the lead contact upon request.

## References

[bib1] Alkan H.F., Walter K.E., Luengo A., Madreiter-Sokolowski C.T., Stryeck S., Lau A.N., Al-Zoughbi W., Lewis C.A., Thomas C.J., Hoefler G. (2018). Cytosolic aspartate availability determines cell survival when glutamine is limiting. Cell Metab..

[bib2] Bakkenist C.J., Kastan M.B. (2004). Initiating cellular stress responses. Cell.

[bib3] Bellier J., Nokin M.J., Caprasse M., Tiamiou A., Blomme A., Scheijen J.L., Koopmansch B., MacKay G.M., Chiavarina B., Costanza B. (2020). Methylglyoxal scavengers resensitize KRAS-mutated colorectal tumors to cetuximab. Cell Rep..

[bib4] Bol V., Bol A., Bouzin C., Labar D., Lee J.A., Janssens G., Porporato P.E., Sonveaux P., Feron O., Grégoire V. (2015). Reprogramming of tumor metabolism by targeting mitochondria improves tumor response to irradiation. Acta Oncol..

[bib5] Boudreau A., Purkey H.E., Hitz A., Robarge K., Peterson D., Labadie S., Kwong M., Hong R., Gao M., Del Nagro C. (2016). Metabolic plasticity underpins innate and acquired resistance to LDHA inhibition. Nat. Chem. Biol..

[bib6] Budczies J., Denkert C., Müller B.M., Brockmöller S.F., Klauschen F., Györffy B., Dietel M., Richter-Ehrenstein C., Marten U., Salek R.M. (2012). Remodeling of central metabolism in invasive breast cancer compared to normal breast tissue - a GC-TOFMS based metabolomics study. BMC Genomics.

[bib7] Carpenter B., Gelman A., Hoffman M.D., Lee D., Goodrich B., Betancourt M., Brubaker M., Guo J., Li P., Riddell A. (2017). *Stan*: a probabilistic programming language. J. Stat. Softw..

[bib8] Chandel N.S. (2015). Evolution of mitochondria as signaling organelles. Cell Metab..

[bib9] Christen S., Lorendeau D., Schmieder R., Broekaert D., Metzger K., Veys K., Elia I., Buescher J.M., Orth M.F., Davidson S.M. (2016). Breast cancer-derived lung metastases show increased pyruvate carboxylase-dependent anaplerosis. Cell Rep..

[bib10] Coleman M.C., Asbury C.R., Daniels D., Du J., Aykin-Burns N., Smith B.J., Li L., Spitz D.R., Cullen J.J. (2008). 2-Deoxy-d-glucose causes cytotoxicity, oxidative stress, and radiosensitization in pancreatic cancer. Free Radic. Biol. Med..

[bib11] Crokart N., Radermacher K., Jordan B.F., Baudelet C., Cron G.O., Grégoire V., Beghein N., Bouzin C., Feron O., Gallez B. (2005). Tumor radiosensitization by antiinflammatory drugs: evidence for a new mechanism involving the oxygen effect. Cancer Res..

[bib12] DeBerardinis R.J., Chandel N.S. (2016). Fundamentals of cancer metabolism. Sci. Adv..

[bib13] Desai R., East D.A., Hardy L., Faccenda D., Rigon M., Crosby J., Alvarez M.S., Singh A., Mainenti M., Hussey L.K. (2020). Mitochondria form contact sites with the nucleus to couple prosurvival retrograde response. Sci. Adv..

[bib14] Elia I., Doglioni G., Fendt S.M. (2018). Metabolic hallmarks of metastasis formation. Trends Cell Biol..

[bib15] Faubert B., Solmonson A., DeBerardinis R.J. (2020). Metabolic reprogramming and cancer progression. Science.

[bib16] Floberg J.M., Schwarz J.K. (2019). Manipulation of glucose and hydroperoxide metabolism to improve radiation response. Semin. Radiat. Oncol..

[bib17] Fouquerel E., Goellner E.M., Yu Z., Gagné J.-P., Barbi de Moura M., Feinstein T., Wheeler D., Redpath P., Li J., Romero G. (2014). ARTD1/PARP1 negatively regulates glycolysis by inhibiting hexokinase 1 independent of NAD+ depletion. Cell Rep..

[bib18] Franken N.A.P., Rodermond H.M., Stap J., Haveman J., van Bree C. (2006). Clonogenic assay of cells in vitro. Nat. Protoc..

[bib19] Fregene A.O. (1967). Calibration of the ferrous sulfate dosimeter by ionometric and calorimetric methods for radiations of a wide range of energy. Radiat. Res..

[bib20] Gabry J., Andreae M., Betancourt M., Carpenter B., Gao Y., Gelman A., Goodrich B., Lee D., Song D., Trangucci R. (2018). shinystan: interactive visual and numerical diagnostics and pos-terior analysis for Bayesian models. https://mc-stan.org/shinystan/.

[bib21] Gallez B., Neveu M.-A., Danhier P., Jordan B.F. (2017). Manipulation of tumor oxygenation and radiosensitivity through modification of cell respiration. A critical review of approaches and imaging biomarkers for therapeutic guidance. Biochim. Biophys. Acta Bioenerg..

[bib22] Goodrich B., Gabry J., Ali I., Brilleman S. (2018). 'rstanarm: Bayesian applied regression modeling via Stan. https://mc-stan.org/rstanarm/.

[bib23] Götting I., Jendrossek V., Matschke J. (2020). A new twist in protein kinase b/akt signaling: role of altered cancer cell metabolism in akt-mediated therapy resistance. Int. J. Mol. Sci..

[bib24] Hähnel P.S., Enders B., Sasca D., Roos W.P., Kaina B., Bullinger L., Theobald M., Kindler T. (2014). Targeting components of the alternative NHEJ pathway sensitizes KRAS mutant leukemic cells to chemotherapy. Blood.

[bib25] Hanahan D., Weinberg R.A. (2011). Hallmarks of cancer: the next generation. Cell.

[bib26] Hlouschek J., Hansel C., Jendrossek V., Matschke J. (2018). The mitochondrial citrate carrier (SLC25A1) sustains redox homeostasis and mitochondrial metabolism supporting radioresistance of cancer cells with tolerance to cycling severe hypoxia. Front. Oncol..

[bib27] Hlouschek J., Ritter V., Wirsdörfer F., Klein D., Jendrossek V., Matschke J. (2018). Targeting SLC25A10 alleviates improved antioxidant capacity and associated radioresistance of cancer cells induced by chronic-cycling hypoxia. Cancer Lett..

[bib28] Hopfner K.-P., Karcher A., Shin D.S., Craig L., Arthur L.M., Carney J.P., Tainer J.A. (2004). Structural biology of Rad50 ATPase. Cell.

[bib29] Krall A.S., Xu S., Graeber T.G., Braas D., Christofk H.R. (2016). Asparagine promotes cancer cell proliferation through use as an amino acid exchange factor. Nat. Commun..

[bib30] Krause M., Brüne M., Theiss C. (2016). Preparation of human formalin-fixed brain slices for electron microscopic investigations. Ann. Anat..

[bib31] Lahiguera Á., Hyroššová P., Figueras A., Garzón D., Moreno R., Soto-Cerrato V., McNeish I., Serra V., Lazaro C., Barretina P. (2020). Tumors defective in homologous recombination rely on oxidative metabolism: relevance to treatments with PARP inhibitors. EMBO Mol. Med..

[bib32] Levi S., Rovida E. (2009). The role of iron in mitochondrial function. Biochim. Biophys. Acta.

[bib33] Li H., Liu Y., Shang L., Cai J., Wu J., Zhang W., Pu X., Dong W., Qiao T., Li K. (2019). Iron regulatory protein 2 modulates the switch from aerobic glycolysis to oxidative phosphorylation in mouse embryonic fibroblasts. Proc. Natl. Acad. Sci. U S A.

[bib34] Liemburg-Apers D.C., Willems P.H.G.M., Koopman W.J.H., Grefte S. (2015). Interactions between mitochondrial reactive oxygen species and cellular glucose metabolism. Arch. Toxicol..

[bib35] Liu R., Fan M., Candas D., Qin L., Zhang X., Eldridge A., Zou J.X., Zhang T., Juma S., Jin C. (2015). CDK1-mediated SIRT3 activation enhances mitochondrial function and tumor radioresistance. Mol. Cancer Ther..

[bib36] Lozoya O.A., Martinez-Reyes I., Wang T., Grenet D., Bushel P., Li J., Chandel N., Woychik R.P., Santos J.H. (2018). Mitochondrial nicotinamide adenine dinucleotide reduced (NADH) oxidation links the tricarboxylic acid (TCA) cycle with methionine metabolism and nuclear DNA methylation. PLoS Biol..

[bib37] Lu C.-L., Qin L., Liu H.-C., Candas D., Fan M., Li J.J. (2015). Tumor cells switch to mitochondrial oxidative phosphorylation under radiation via mTOR-mediated hexokinase II inhibition - a Warburg-reversing effect. PLoS One.

[bib38] Matschke J., Riffkin H., Klein D., Handrick R., Lüdemann L., Metzen E., Shlomi T., Stuschke M., Jendrossek V. (2016). Targeted inhibition of glutamine-dependent glutathione metabolism overcomes death resistance induced by chronic cycling hypoxia. Antioxid. Redox Signal..

[bib39] Mladenov E., Magin S., Soni A., Iliakis G. (2013). DNA double-strand break repair as determinant of cellular radiosensitivity to killing and target in radiation therapy. Front. Oncol..

[bib40] Mohanti B.K., Rath G.K., Anantha N., Kannan V., Das B.S., Chandramouli B.A., Banerjee A.K., Das S., Jena A., Ravichandran R. (1996). Improving cancer radiotherapy with 2-deoxy-d-glucose: phase I/II clinical trials on human cerebral gliomas. Int. J. Radiat. Oncol. Biol. Phys..

[bib41] Morandi A., Indraccolo S. (2017). Linking metabolic reprogramming to therapy resistance in cancer. Biochim. Biophys. Acta Rev. Cancer.

[bib42] Murphy M.P., Smith R.A.J. (2007). Targeting antioxidants to mitochondria by conjugation to lipophilic cations. Annu. Rev. Pharmacol. Toxicol..

[bib43] Nicoletti I., Migliorati G., Pagliacci M.C., Grignani F., Riccardi C. (1991). A rapid and simple method for measuring thymocyte apoptosis by propidium iodide staining and flow cytometry. J. Immunol. Methods.

[bib44] Oeck S., Malewicz N.M., Hurst S., Al-Refae K., Krysztofiak A., Jendrossek V. (2017). The focinator v2-0 – graphical interface, four channels, colocalization analysis and cell phase identification. Radiat. Res..

[bib45] Oeck S., Malewicz N.M., Hurst S., Rudner J., Jendrossek V. (2015). The Focinator - a new open-source tool for high-throughput foci evaluation of DNA damage. Radiat. Oncol..

[bib46] Oexle H., Gnaiger E., Weiss G. (1999). Iron-dependent changes in cellular energy metabolism: influence on citric acid cycle and oxidative phosphorylation. Biochim. Biophys. Acta.

[bib47] Paul B.T., Manz D.H., Torti F.M., Torti S.V. (2017). Mitochondria and iron: current questions. Expert Rev. Hematol..

[bib48] Paull T.T., Gellert M. (1999). Unwinding and endonuclease cleavage by the Mre11/Rad50 complex. Genes Dev..

[bib49] Petrat F., Weisheit D., Lensen M., De Groot H., Sustmann R., Rauen U. (2002). Selective determination of mitochondrial chelatable iron in viable cells with a new fluorescent sensor. Biochem. J..

[bib50] Pouliliou S., Koukourakis M.I. (2014). Gamma histone 2AX (**γ**-H2AX)as a predictive tool in radiation oncology. Biomarkers.

[bib51] Prasanna V.K., Venkataramana N.K., Dwarakanath B.S., Santhosh V. (2009). Differential responses of tumors and normal brain to the combined treatment of 2-DG and radiation in glioablastoma. J. Cancer Res. Ther..

[bib52] Qin L., Fan M., Candas D., Jiang G., Papadopoulos S., Tian L., Woloschak G., Grdina D.J., Li J.J. (2015). CDK1 enhances mitochondrial bioenergetics for radiation-induced DNA repair. Cell Rep..

[bib53] Qu J., Sun W., Zhong J., Lv H., Zhu M., Xu J., Jin N., Xie Z., Tan M., Lin S.-H. (2017). Phosphoglycerate mutase 1 regulates dNTP pool and promotes homologous recombination repair in cancer cells. J. Cell Biol..

[bib54] Rashmi R., Huang X., Floberg J.M., Elhammali A.E., McCormick M.L., Patti G.J., Spitz D.R., Schwarz J.K. (2018). Radioresistant cervical cancers are sensitive to inhibition of glycolysis and redox metabolism. Cancer Res..

[bib55] Reznik E., Luna A., Aksoy B.A., Liu E.M., La K., Ostrovnaya I., Creighton C.J., Hakimi A.A., Sander C. (2018). A landscape of metabolic variation across tumor types. Cell Syst..

[bib56] Roos W.P., Thomas A.D., Kaina B. (2015). DNA damage and the balance between survival and death in cancer biology. Nat. Rev. Cancer.

[bib57] Rouschop K.M., Dubois L.J., Keulers T.G., Van Den Beucken T., Lambin P., Bussink J., Van Der Kogel A.J., Koritzinsky M., Wouters B.G. (2013). PERK/eIF2α signaling protects therapy resistant hypoxic cells through induction of glutathione synthesis and protection against ROS. Proc. Natl. Acad. Sci. U S A.

[bib58] Sanli T., Rashid A., Liu C., Harding S., Bristow R.G., Cutz J.-C., Singh G., Wright J., Tsakiridis T. (2010). Ionizing radiation activates AMP-activated kinase (AMPK): a target for radiosensitization of human cancer cells. Int. J. Radiat. Oncol..

[bib59] Santambrogio P., Dusi S., Guaraldo M., Rotundo L.I., Broccoli V., Garavaglia B., Tiranti V., Levi S. (2015). Mitochondrial iron and energetic dysfunction distinguish fibroblasts and induced neurons from pantothenate kinase-associated neurodegeneration patients. Neurobiol. Dis..

[bib60] Shackelford D.B., Abt E., Gerken L., Vasquez D.S., Seki A., Leblanc M., Wei L., Fishbein M.C., Czernin J., Mischel P.S., Shaw R.J. (2013). LKB1 inactivation dictates therapeutic response of non-small cell lung cancer to the metabolism drug phenformin. Cancer Cell.

[bib61] Simpson-Lavy K.J., Bronstein A., Kupiec M., Johnston M. (2015). Cross-talk between carbon metabolism and the DNA damage response in *S. cerevisiae*. Cell Rep..

[bib62] Son J., Lyssiotis C.A., Ying H., Wang X., Hua S., Ligorio M., Perera R.M., Ferrone C.R., Mullarky E., Shyh-Chang N. (2013). Glutamine supports pancreatic cancer growth through a KRAS-regulated metabolic pathway. Nature.

[bib63] Sondka Z., Bamford S., Cole C.G., Ward S.A., Dunham I., Forbes S.A. (2018). The COSMIC Cancer Gene Census: describing genetic dysfunction across all human cancers. Nat. Rev. Cancer.

[bib64] Sonveaux P., Végran F., Schroeder T., Wergin M.C., Verrax J., Rabbani Z.N., De Saedeleer C.J., Kennedy K.M., Diepart C., Jordan B.F. (2008). Targeting lactate-fueled respiration selectively kills hypoxic tumor cells in mice. J. Clin. Invest..

[bib65] Tang L., Wei F., Wu Y., He Y., Shi L., Xiong F., Gong Z., Guo C., Li Xiayu, Deng H. (2018). Role of metabolism in cancer cell radioresistance and radiosensitization methods. J. Exp. Clin. Cancer Res..

[bib66] Toulany M., Iida M., Keinath S., Iyi F.F., Mueck K., Fehrenbacher B., Mansour W.Y., Schaller M., Wheeler D.L., Rodemann H.P. (2016). Dual targeting of PI3K and MEK enhances the radiation response of K-RAS mutated non-small cell lung cancer. Oncotarget.

[bib67] Turgeon M.-O., Perry N.J.S., Poulogiannis G. (2018). DNA damage, repair, and cancer metabolism. Front. Oncol..

[bib68] Vega-Rubin-de-Celis S., Abdallah Z., Kinch L., Grishin N.V., Brugarolas J., Zhang X. (2010). Structural analysis and functional implications of the negative mTORC1 regulator REDD1. Biochemistry.

[bib69] Vehtari A., Gelman A., Gabry J. (2017). Practical Bayesian model evaluation using leave-one-out cross-validation and WAIC. Stat. Comput..

[bib70] Viale A., Pettazzoni P., Lyssiotis C.A., Ying H., Sánchez N., Marchesini M., Carugo A., Green T., Seth S., Giuliani V. (2014). Oncogene ablation-resistant pancreatic cancer cells depend on mitochondrial function. Nature.

[bib71] Weinberg S.E., Chandel N.S. (2015). Targeting mitochondria metabolism for cancer therapy. Nat. Chem. Biol..

[bib72] Wu Z., Oeck S., West A.P., Mangalhara K.C., Sainz A.G., Newman L.E., Zhang X.O., Wu L., Yan Q., Bosenberg M. (2019). Mitochondrial DNA stress signalling protects the nuclear genome. Nat. Metab..

[bib73] Xu W., Barrientos T., Andrews N.C. (2013). Iron and copper in mitochondrial diseases. Cell Metab..

[bib74] Yang Y., Luo M., Zhang K., Zhang J., Gao T., Connell D.O., Yao F., Mu C., Cai B., Shang Y., Chen W. (2020). Nedd4 ubiquitylates VDAC2/3 to suppress erastin-induced ferroptosis in melanoma. Nat. Commun..

[bib75] Yuneva M.O., Fan T.W.M., Allen T.D., Higashi R.M., Ferraris D.V., Tsukamoto T., Matés J.M., Alonso F.J., Wang C., Seo Y. (2012). The metabolic profile of tumors depends on both the responsible genetic lesion and tissue type. Cell Metab..

[bib76] Zhou Z., Ye T.J., DeCaro E., Buehler B., Stahl Z., Bonavita G., Daniels M., You M. (2020). Intestinal SIRT1 deficiency protects mice from ethanol-induced liver injury by mitigating ferroptosis. Am. J. Pathol..

