# Supplementary material for: Metabolism of cancer cells commonly responds to irradiation by a transient early mitochondrial shutdown
Source: iScience. 2021 Oct 28;24(11):103366. doi: 10.1016/j.isci.2021.103366 (PMC8603201; doi:10.1016/j.isci.2021.103366)
Supplement: Document S1. Figures S1–S9 and Table S1 [file mmc1.pdf]

iScience, Volume *24*

Supplemental information

Metabolism of cancer cells commonly responds to irradiation by a transient early mitochondrial shutdown

Adam Krysztofiak, Klaudia Szymonowicz, Julian Hlouschek, Kexu Xiang, Christoph Waterkamp, Safa Larafa, Isabell Goetting, Silvia Vega-Rubin-de-Celis, Carsten Theiss, Veronika Matschke, Daniel Hoffmann, Verena Jendrossek, and Johann Matschke

Figure S1. (Related to Figure 1)

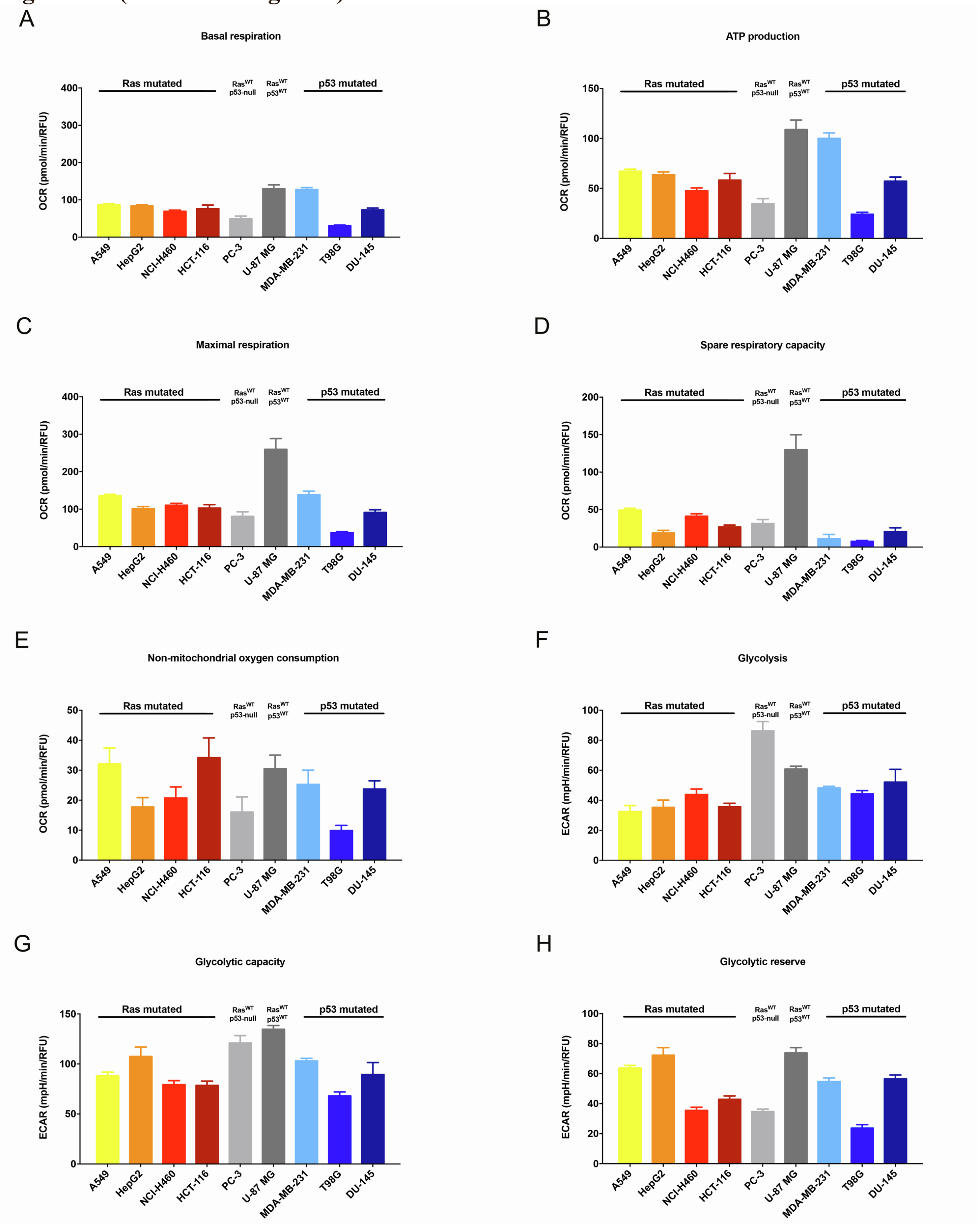

Figure S1. (Related to Figure 1) Metabolic parameters from extracellular flux assays for experimental cell lines panel.

(A-D) Extracellular flux assay analysis showing excessive heterogeneity in metabolic parameters across different tumor entities without IR. Cancer cell lines represent diverse levels of basal respiration (A), ATP production (B), maximal respiration (C), spare respiratory capacity (D), non-mitochondrial oxygen consumption (E), Glycolysis (F), glycolytic capacity (G) and glycolytic reserve (H). Data show mean values ± SEM from n=8-16 wells for each cell lines, combined from N=2 independent experiments. Each value characterizes oxygen consumption rate (OCR – A, B) or ECAR (C, D) and was normalized to cell number at the end of the assay, defined by a DNA-content staining using Hoechst33342 (pmol/min/RFU).

Figure S2. (Related to Figure 2)

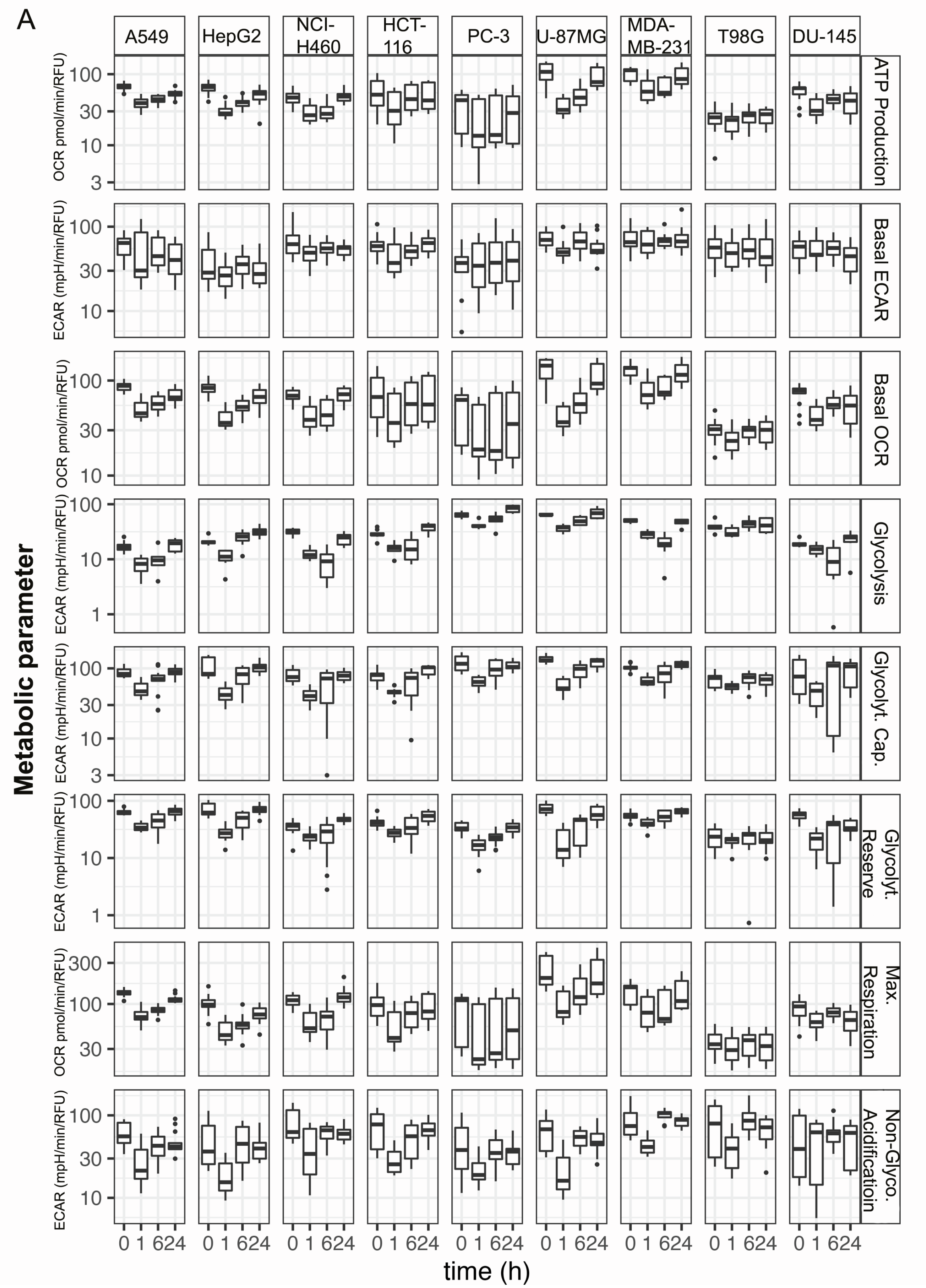

Figure S2. (Related to Figure 2) Changes of metabolic parameters over time after IR-treatment.

(A) Oxygen consumption rate (OCR) and extracellular acidification rate (ECAR) values for the measured metabolic parameters (y-axis) of non-irradiated cancer cells (0h), and at 1, 6, 24h after irradiation with 3Gy as indicated. Tukey-boxplots with whiskers marking the quartiles of measured values. Plotted OCR or ECAR values were obtained from n=8-16 wells for each cell line, combined from N=2 independent experiments and normalized to cell number at the end of the assay, defined by a DNA-content staining using Hoechst33342 (pmol/min/RFU).

Figure S3. (Related to Figure 2)

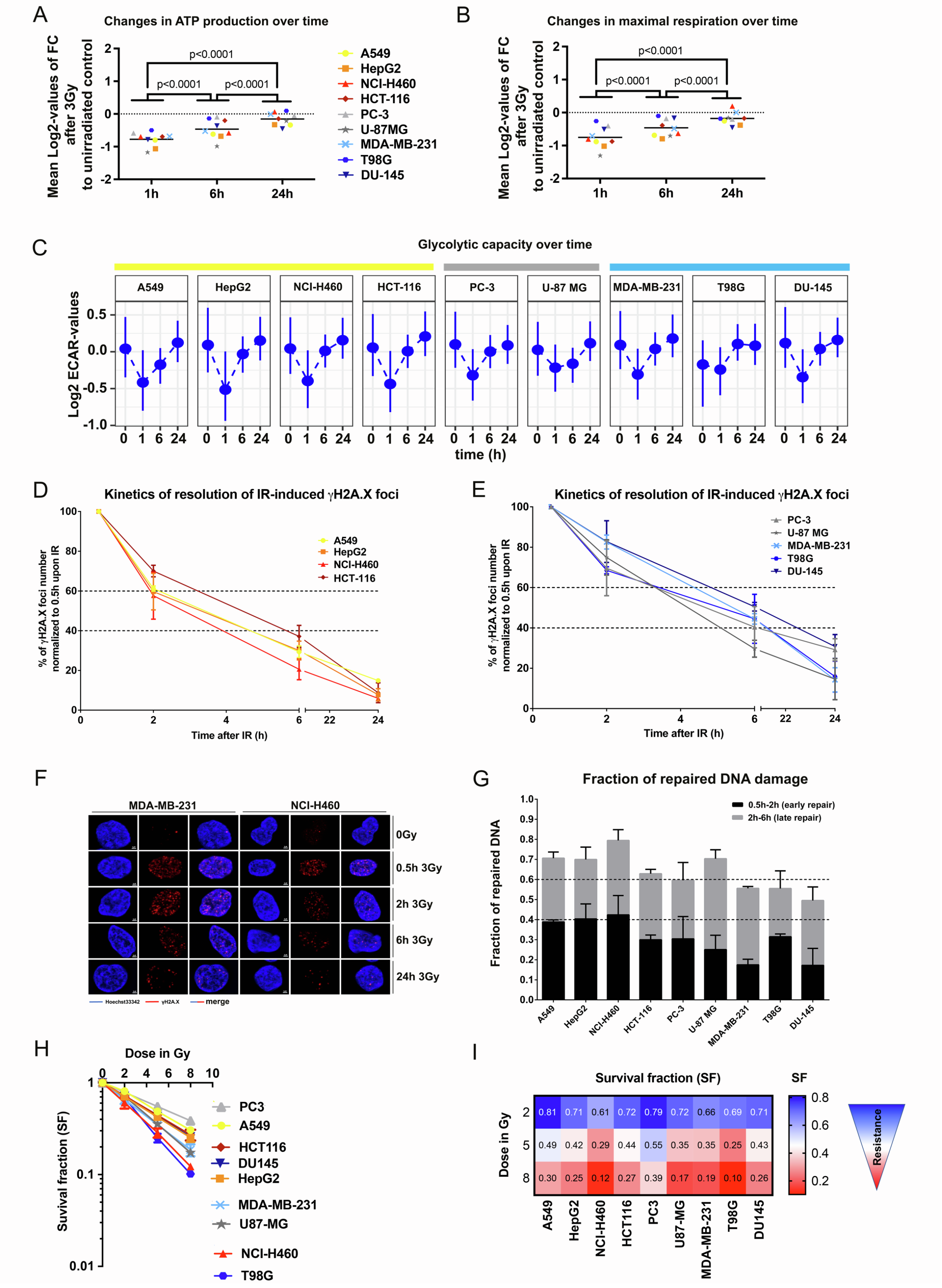

Figure S3. (Related to Figure 2) Effects of IR-treatment on metabolism, DNA repair and survival.

(A-B) Log2-values of changes to measured ATP production (A) and maximal respiration (B) induced by IR with a dose of 3Gy to measured basal respiration compared to non-irradiated controls 1, 6 and 24h after IR are indicated. Mean values of n=12-16 wells per cell line from N=2 independent experiments are indicated, and p-values were calculated using two-way ANOVA with Tukey's multiple comparisons post hoc test. (C) Differences observed in use of glycolysis (expressed as compensatory glycolysis parameter – glycolytic capacity) between Ras and p53 mutated cell lines over time after irradiation with 3Gy. Yellow represents cell lines with Ras mutation, grey with intermixed mutation and blue with p53 mutations. Error bars mark intervals between 5% and 95% quantiles of marginal posterior distribution of log2 ECAR values. (D-E) All tested cell lines were divided into 2 groups (fast (D) and slowly (E) repairing) regarding their IR-induced DNA repair kinetics determined using the γH2A.X assay. Data represent mean % of foci number at indicated time points normalized to 30 min time point ± SEM (N=3). (F) Representative pictures of NCI-H60 and MDA-MB-231 cell lines from γH2A.X assay at indicated time points. Blue – Hoechst33342; red - γH2A.X. Scale bar: 2 µm. (G) Comparison between early (0.5h to 2h) and late (2h to 6h) DNA damage repair speed presented as relative foci number reduction. Data represent mean % of foci number at indicated time points normalized to 30 min time point ± SEM (N=3). (H) Survival curves representing the mean survival fraction (SF) ± SEM of clonogenic survival in 9 cell lines upon IR with 2-8Gy (N=3). (I) Heatmap representing the mean survival fraction (SF) at different IR-doses (2, 5, 8Gy) in all cell lines.

Figure S4. (Related to Figure 2, 3)

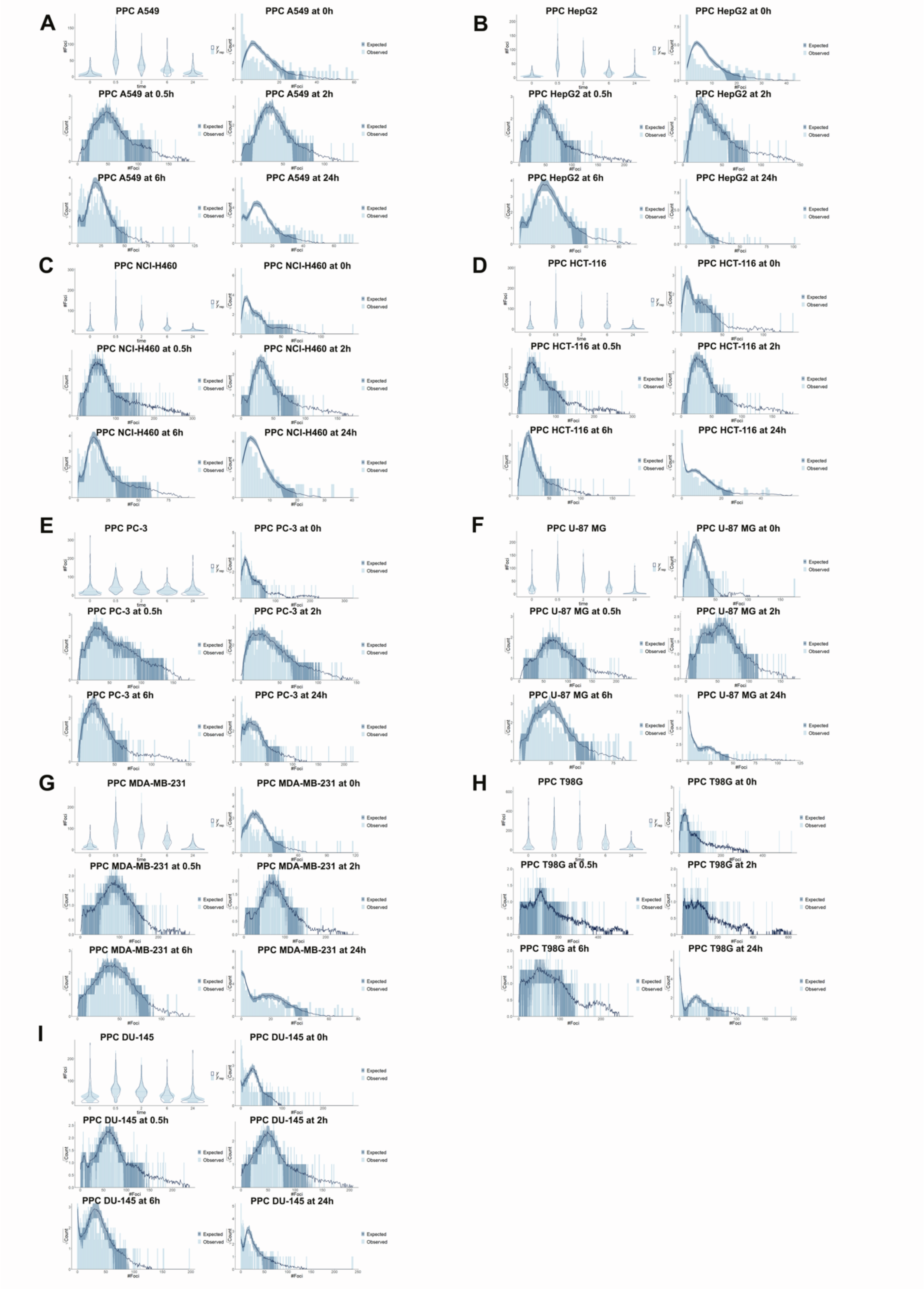

Figure S4. (Related to Figure 2, 3) Validity of the statistical model for expected number of γH2A.X foci at indicated time points in all cell lines.

(A-I) Posterior predictive check (PPC) of the statistical models for numbers of γ-H2A.X foci of 9 tested cancer cell lines (A549 (A), HepG2 (B), NCI-H460 (C), HCT-116 (D), PC-3 (E), U-87 MG (F), MDA-MB-231 (G), T98G (H), DU-145 (I)) as violin plots and as distribution of γH2A.X foci at the indicated time point. Data represent N=3 independent experiments.

Figure S5. (Related to Figure 2)

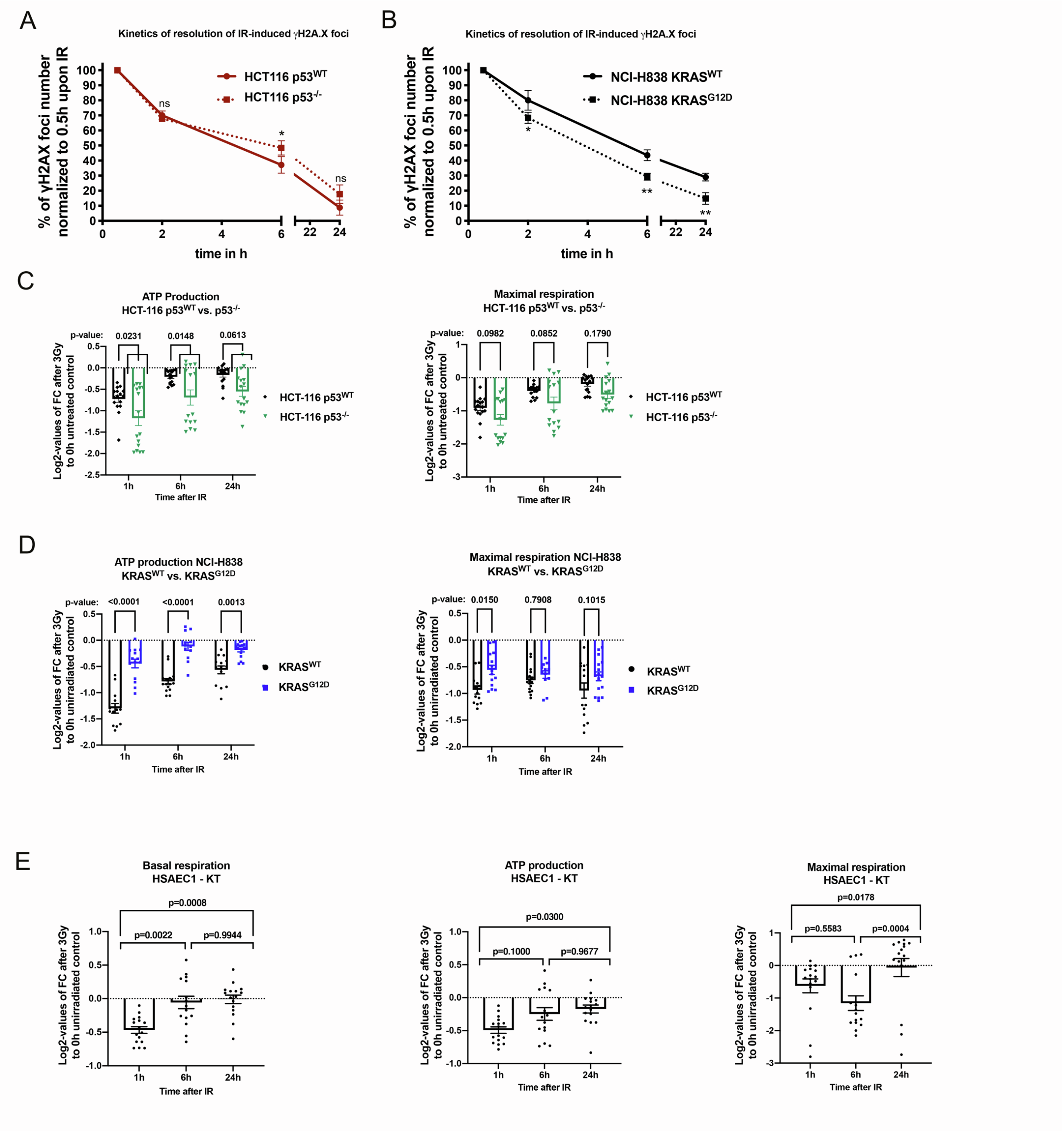

Figure S5. (Related to Figure 2) Influence of oncogenic activation (p53 vs. Ras) on DNA repair and cellular metabolism in irradiated isogenic cancer cell lines.

(A-B) DNA repair kinetics of isogenic cancer cell line pairs determined using the γH2A.X assay after treatment with IR of 3Gy as indicated: HCT-116 p53^{WT} *vs.* HCT-116 p53$^{-/-}$ (A), NCI-H838 KRAS^{WT} vs. NCI-H838 KRAS^{G12D} (B) cell lines. Data represent mean % of foci number at indicated time points normalized to 30min time point ± SEM (N=3). p-values were calculated

using 2-way ANOVA with Šídák's multiple comparisons post hoc test. ns: not significant, * $p<0.05$, ** $p\leq0.01$. (C-E) Figures depict Log2-values of changes to measured basal respiration, ATP production and maximal respiration induced by IR in isogenic cancer cell pairs bearing specific genetic alterations in oncogenes and tumor suppressor genes as indicated and normal lung epithelial cells (HSAEC1-KT) with a dose of 3Gy to compared to non-irradiated controls at 1, 6 and 24h after treatment as follows: (C) HCT-116 $p53^{WT}$ vs. HCT-116 $p53^{-/-}$; (D) NCI-H838 $KRAS^{WT}$ vs. NCI-H838 $KRAS^{G12D}$; (E) HSAEC1-KT). Mean values of n=10-16 wells per cell line from N=2 independent experiments are indicated ± SEM (p-values calculated using two-way ANOVA with Šídák's multiple comparisons post hoc test).

Figure S6. (Related to Figure 3)

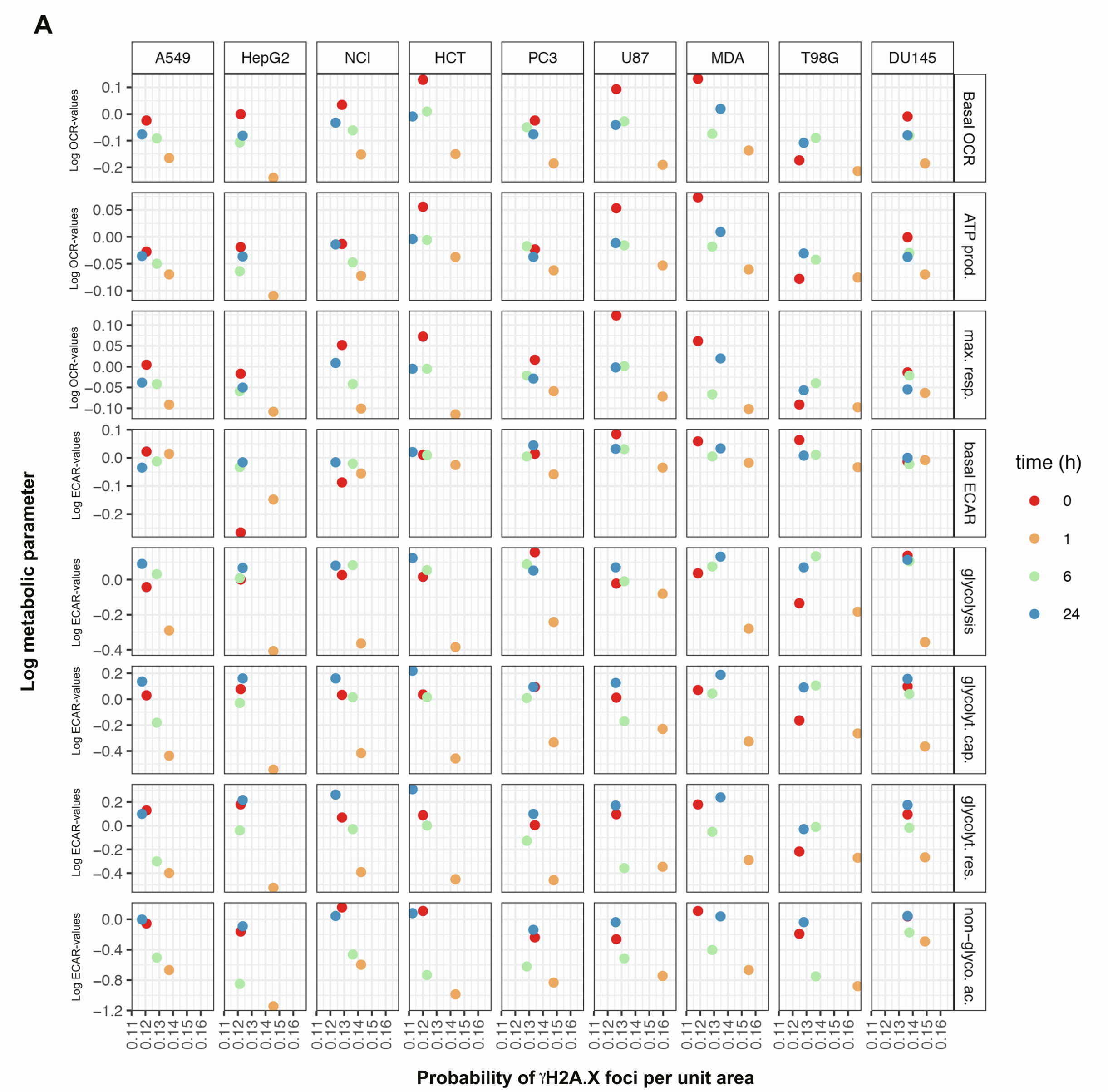

Figure S6. (Related to Figure 3) Correlation of metabolic parameters to DNA repair kinetics.

(A) Log metabolic parameters (y-axis) versus the probability of γH2A.X foci per unit area (0.1*Pixel^2) in cancer cell lines at indicated timepoints after IR with 3Gy, both sets of values predicted by the statistical model fitted to the measurements.

Figure S7. (Related to Figure 3)

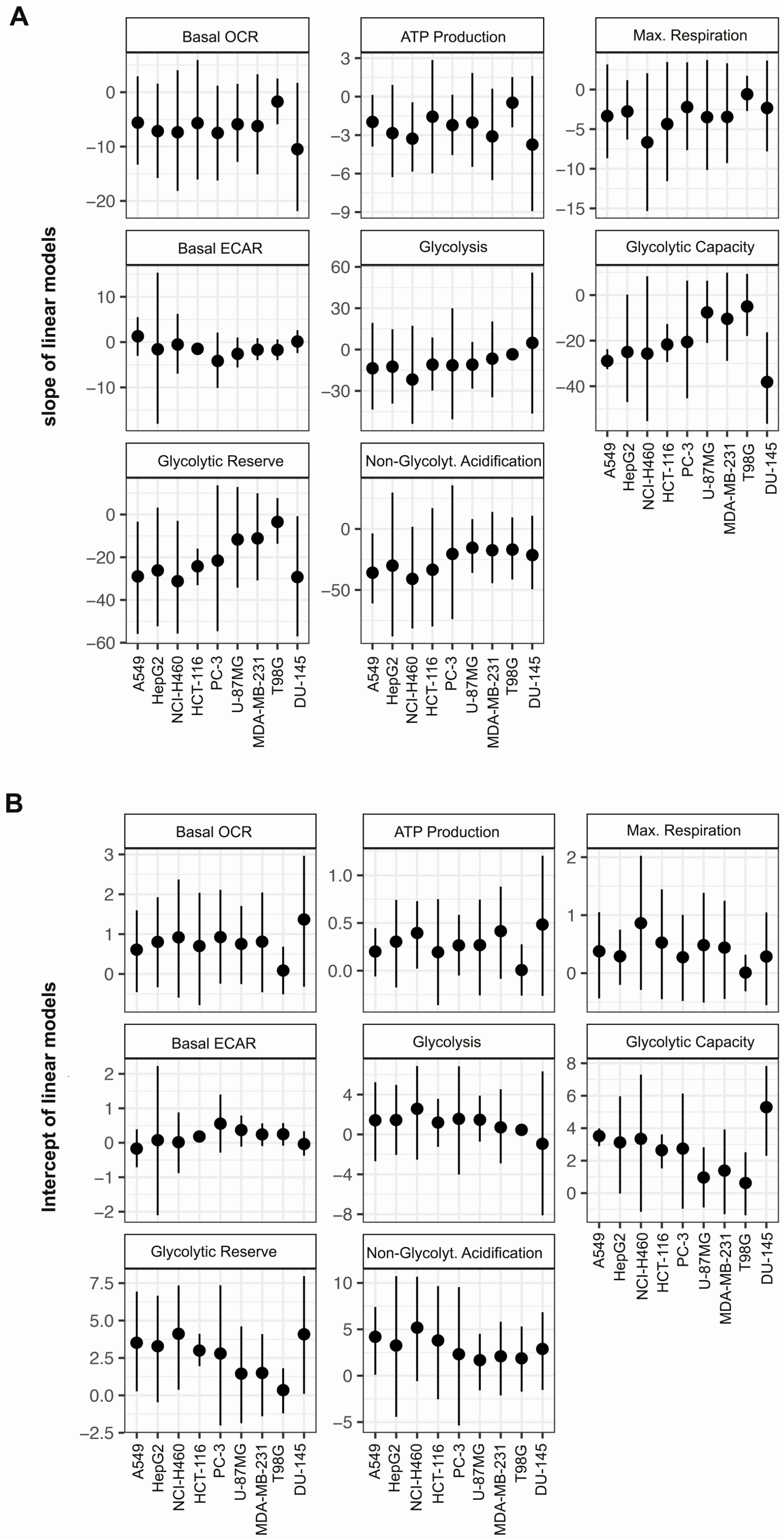

Figure S7. (Related to Figure 3) Slopes and Intercepts of correlated metabolic parameters to DNA repair kinetics.

(A) Probability distributions of slopes of linear models (Figure. 5A) of metabolic parameter and probability of γH2A.X foci per unit area. (B) Probability distributions of Intercepts of same linear models. Error bars mark intervals between 5% and 95% quantiles of marginal posterior distributions of model slopes (A) and intercepts (B).

Figure S8. (Related to Figure 2, 4, and 5)

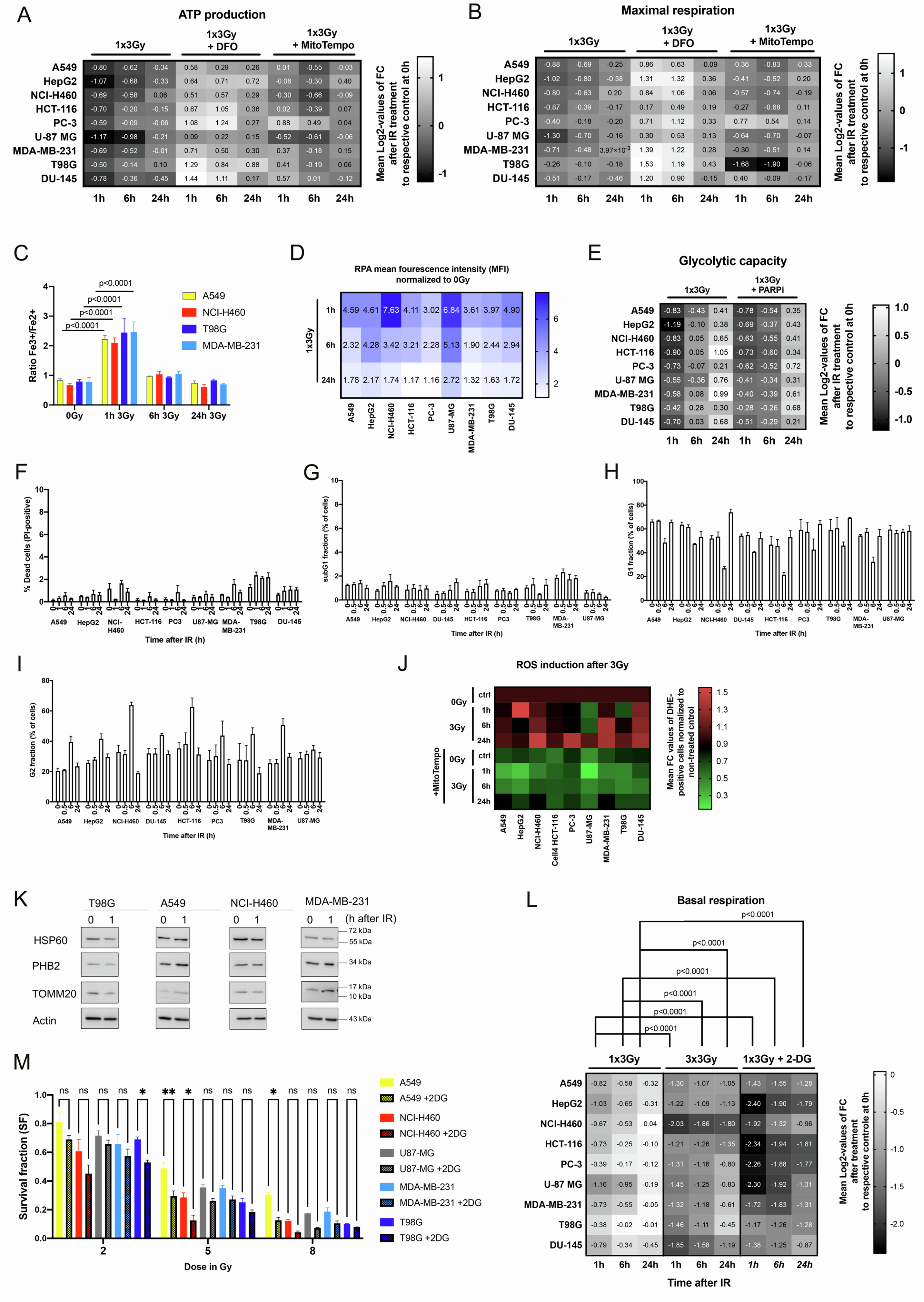

Figure S8. (Related to Figure 2, 4, and 5) Mechanistic basis of mitochondrial shutdown.

(A-B) Mean Log2-values of changes to measured ATP production (A) and maximal respiration (B) induced by IR alone or in combination treatment with DFO (50μM) or MitoTempo (1nM) and IR with a dose of 3Gy to compared to non-irradiated controls 1, 6 and 24h after treatment are indicated. (C) Calculated mean Fe^{3+}/Fe^{2+} ratios ±SD induced by IR with a dose of 3Gy 1, 6 and 24h after IR. Two-way ANOVA with Bonferroni post hoc test. (D) Heatmap representing RPA mean florescence normalized to non-irradiated (0Gy) controls. RPA fluorescence is specifically quenched by Fe^{2+}. (E) Heatmap representing mean Log2-values of changes to measured glycolytic capacity induced by IR with a dose of 3Gy alone or in combination with PARP-inhibitor (PJ34, 4μM) to measured basal glycolytic capacity compared to non-irradiated controls 1, 6 and 24h after IR. (F) Cell death and (G-I) Cell cycle analysis upon IR with 3Gy Mean values ± SEM (N=3) are shown. Fraction of cells (G) with fragmented DNA (SubG1 fraction), (H) in G1-phase and (I) G2-phase of cell cycle in percent (%) in unirradiated cells and 1, 6, and 24h after IR with a dose of 3Gy. (J) Heatmap representing mean ROS induction (relative to non-irradiated controls) by IR with a dose of 3Gy alone or in combination with MitoTempo (1nM) treatment. Mitophagy activity was determined by assessing the levels of the inner mitochondrial membrane mitophagy receptor PHB2 (Prohibitin 2, (Murphy and Smith, 2007)), intramitochondrial HSP60 (heat-shock protein 60) and outer mitochondrial membrane TOMM20 (Translocase of outer mitochondrial membrane 20) 1h after exposure to irradiation. (K) Western blot representing mitophagy markers in untreated (0) and 1h after IR with a dose of 3Gy (L) Mean Log2-values of changes to measured basal respiration induced by IR alone, fractionated dose of 3x 3Gy applied on every 24h for 3 consecutive days or in combination treatment with 2DG and IR with a dose of 3Gy to compared to non-irradiated controls 1, 6 and 24h after treatment are indicated. FC: Fold change; p-values calculated using two-way ANOVA with Tukey's multiple comparisons post hoc test. (M) Mean survival fraction (SF) ± SD at different IR-doses (2, 5, 8Gy) alone or in combination with 2DG treatment. p-values were calculated using 2-way ANOVA with Tukey's multiple comparisons post hoc test. ns: not significant, * $p<0.05$, ** $p\leq0.01$

Figure S9. (Related to Figure 5)

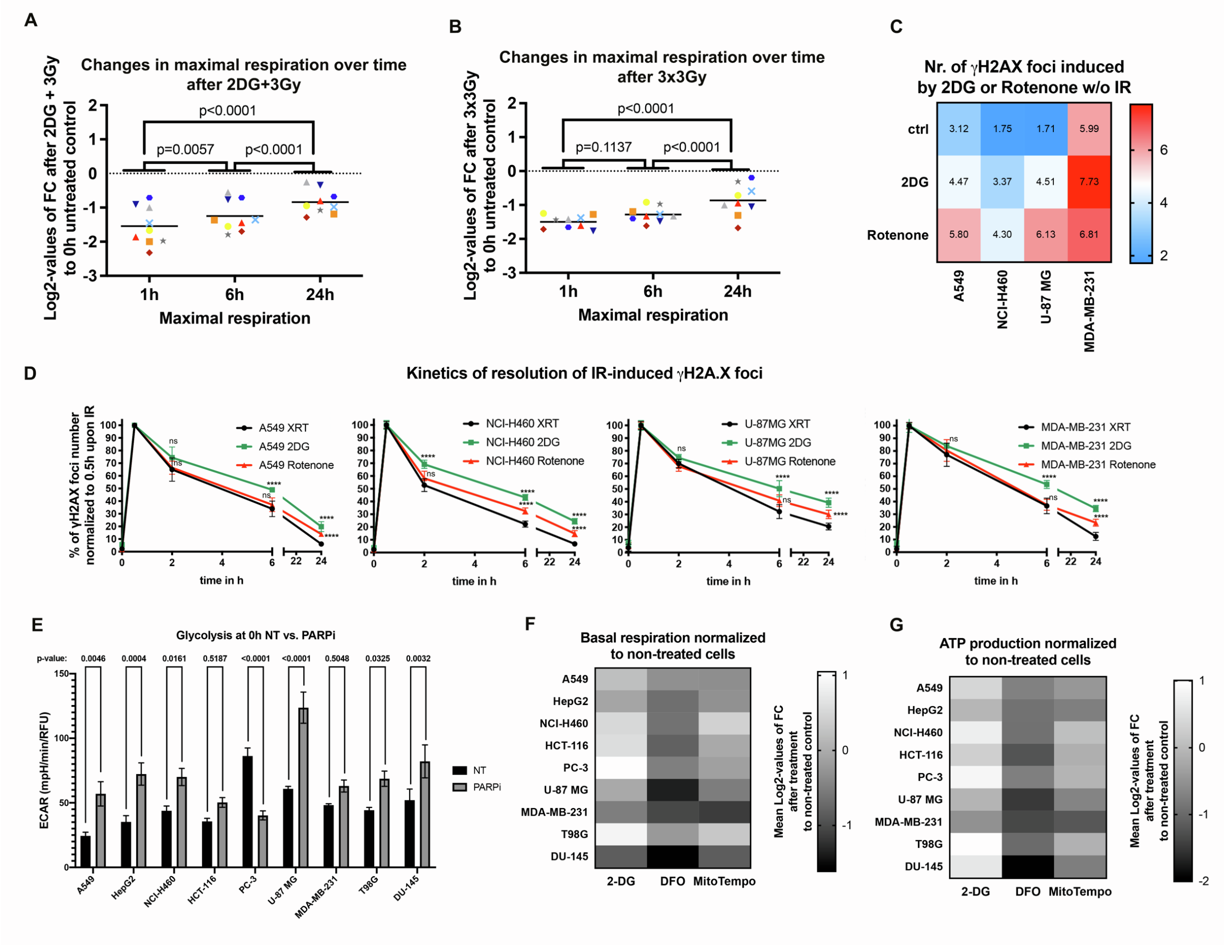

Figure S9. (Related to Figure 5) Effects of Targeting on DNA repair and mitochondrial respiration.

(A) Log2-values of changes to measured maximal respiration induced by combination treatment with 2DG (20mM) and IR with a dose of 3Gy to compared to non-irradiated controls 1, 6 and 24h after treatment are indicated. (B) Log2-values of changes to measured maximal respiration induced by fractionated dose of 3x 3Gy applied on every 24h for 3 consecutive days compared to non-irradiated controls 1, 6 and 24h after treatment are indicated. Mean values of n=8-16 wells per cell line from N=2 independent experiments are indicated. p-values were calculated using 2-way ANOVA with Tukey's multiple comparisons post hoc test. All metabolic parameters were normalized to Hoechst intensity (relative fluorescence units, RFU) in each well. OCR: oxygen consumption rate; ECAR: Extracellular acidification rate; FC: Fold change. (C) number (Nr.) of measured γH2A.X foci upon treatment with 2DG (20mM) or Rotenone (0.8µM) alone for 24 h.

(D) DNA repair kinetics determined using the γH2A.X assay after treatment with IR of 3Gy alone or in combination with 2DG (20 mM) or Rotenone (0.8 µM). Data represent mean % of foci number at indicated time points normalized to 30 min time point ± SEM (N=3). p-values were calculated using 2-way ANOVA with Tukey's multiple comparisons post hoc test. ns: not significant, **** $p \leq 0.0001$. (E) EACR values normalized to cell number of non-treated (NT) and PARP-inhibitor (PARPi, PJ34 (4µM) representing the glycolysis in indicated cell lines. Mean values ± SEM are shown. p-values were calculated using 2-way ANOVA with Tukey's multiple comparisons post hoc test. (F-G) Mean Log2-values of changes to basal respiration (F) and ATP production (G) induced by DFO (50µM) or MitoTempo (1nM) treatment alone compared to non-treated controls.

Table S1. (Related to STAR Methods) Extracellular flux assay conditions.

List of extracellular flux assay conditions used throughout the manuscript (Oligomycin, FCCP, Rotenone, Antimycin A concentrations), cell densities and descriptions of extracellular flux assay parameter calculations.

Extracellular flux assay conditions and cell density

							(cells/well)	
**Cell line**	**Oligomycin**	**FCCP**	**Rotenon**	**Antymycin A**	**glucose**	**2-deoxyglucose**	**24h seeding**	**48h seeding**
**A549**	1 µM	1 µM	0.5 µM	0.5 µM	10 µM	50 µM	10,000	7,500
**HepG2**	1 µM	1 µM	0.5 µM	0.5 µM	10 µM	50 µM	7,500	5,000
**NCI-H460**	1 µM	2 µM	0.5 µM	0.5 µM	10 µM	50 µM	10,000	7,500
**HCT-116 $p53^{WT}$**	1 µM	0.5 µM	0.5 µM	0.5 µM	10 µM	50 µM	10,000	7,500
**PC-3**	1 µM	1 µM	0.5 µM	0.5 µM	10 µM	50 µM	7,500	5,000
**U-87 MG**	1 µM	2 µM	0.5 µM	0.5 µM	10 µM	50 µM	12,500	10,000
**MDA-MB-231**	1 µM	1 µM	0.5 µM	0.5 µM	10 µM	50 µM	7,500	5,000
**T98G**	1 µM	2 µM	0.5 µM	0.5 µM	10 µM	50 µM	10,000	7,500
**DU-145**	1 µM	1.5 µM	0.5 µM	0.5 µM	10 µM	50 µM	12,500	10,000
**NCI-H838 $KRAS^{WT}$**	1µM	1µM	0.5µM	0.5µM	10µM	50µM	10000	7500
**NCI-H838 $KRAS^{G12D}$**	1µM	1µM	0.5µM	0.5µM	10µM	50µM	10000	7500
**HCT-116 $p53^{-/-}$**	1 µM	0.5 µM	0.5 µM	0.5 µM	10 µM	50 µM	10000	7500
**HSAEC1-KT**	1 µM	1.0 µM	0.5 µM	0.5 µM	10 µM	50 µM	20000	20000

Extracellular flux assay parameters calculations - Mito Stress test

**Parameter**	**Definition**
Basal Respiration	(Last rate measurement before first injection) – (Non-Mitochondrial Respiration Rate)
ATP Production	(Last rate measurement before Oligomycin injection) – (Minimum rate measurement after Oligomycin injection)
Maximal Respiration	(Maximum rate measurement after FCCP injection) – (Non-Mitochondrial Respiration)
Spare respiratory capacity	(Maximal Respiration) – (Basal Respiration)
Non-mito.Oxygen Consumption	(Minimum rate measurement after Rotenone/antimycin A injection)

Extracellular flux assay parameters calculations - Glycoliysis Stress test

**Parameter**	**Definition**
Glycolysis	(Maximum rate measurement before Oligomycin injection) – (Last rate measurement before Glucose injection)
Glycolytic Capacity	(Maximum rate measurement after Oligomycin injection) – (Last rate measurement before Glucose injection)
Glycolytic Reserve	(Glycolytic Capacity) – (Glycolysis)
Non-mito.Oxygen Consumption	Last rate measurement before Glucose injection
